# Therapeutic potential of *Echinocactus grusonii* and its associated endophytic fungus *Aspergillus oryzae* in neuroprotection: in vitro and in silico investigations

**DOI:** 10.1038/s41598-026-62565-5

**Published:** 2026-07-29

**Authors:** Ahmed A. Heraiz, Ahmed Othman, Amr Farouk, Mostafa M. Hegazy, Abd El-Salam I. Mohammed, Atef A. El-Hela

**Affiliations:** 1 Department of Pharmacognosy and Medicinal Plants, Faculty of Pharmacy, Al-Azhar University, Cairo, 11884 Egypt; 2 Flavour and Aroma Chemistry Department, National Research Centre, Dokki, Giza, 12622 Egypt

**Keywords:** Cactaceae, *Kroenleinia grusonii*, Neuroprotection, Computational studies, Liquid chromatography–mass spectrometry (LC–MS), Bioactive metabolites, Biochemistry, Biotechnology, Drug discovery, Microbiology, Plant sciences

## Abstract

The Golden Barrel Cactus (*Echinocactus grusonii*, syn. *Kroenleinia grusonii*), native to Mexico, is well known in horticulture but remains insufficiently studied with respect to its chemical composition. This study examined the chemical profile and neuroprotective potential of *Echinocactus grusonii* spines and its endophytic fungus, *Aspergillus oryzae*. The spines contained markedly higher levels of phenolics (293 mg gallic acid/g DW extract), flavonoids (132 mg quercetin/g DW extract), and alkaloids (14.5 mg atropine/g DW extract) compared to the stem. Untargeted metabolomics (UPLC-HRMS/MS) tentatively identified 27 metabolites in the spines and 14 in the fungal extract. Both extracts exhibited potent, dose-dependent inhibition of acetylcholinesterase (AChE) and *β*-secretase (BACE-1). The spines extract showed IC₅₀ values of 0.362 µg/mL (AChE) and 0.425 µg/mL (BACE-1), while the fungal extract was more effective against AChE (IC₅₀ = 0.255 µg/mL). In silico docking was performed for all identified compounds, and molecular dynamics simulation of the top-scoring candidates demonstrated stable interactions with the target enzymes. These findings highlight the spines of *Echinocactus grusonii* and their associated endophytes as promising dual-target neuroprotective sources for the management of neurodegenerative disorders.

**Supplementary Information:**

The online version contains supplementary material available at 10.1038/s41598-026-62565-5.

## Introduction

Cacti (family *Cactaceae*) are succulent xerophytes of considerable ecological and economic importance, particularly in the arid regions of the Americas^1^. To withstand extreme environmental conditions, they have evolved distinctive adaptations, including spines derived from modified leaves, Crassulacean acid metabolism (CAM), and water-storing photosynthetic stems^2,3^. Beyond their role in physical defence and reducing solar irradiation, spines also contribute to plant’s survival by hosting microorganisms that may facilitate microbial-mediated deterrence of herbivores^4^. Moreover, cacti represent a rich source of secondary metabolites—such as alkaloids, flavonoids, and terpenoids—which exhibit neuroactive, anti-inflammatory, and antioxidant properties^2^.

*Echinocactus grusonii* Hildm. (*Kroenleinia grusonii*), commonly known as the Golden Barrel Cactus, is a member of the Cactaceae family native to Mexico. Previous investigations of its stems have led to the isolation of compounds exhibiting notable neuroprotective activity^1,5^. In parallel, endophytic fungi inhabiting desert cacti play critical ecological roles, enhancing host tolerance to heat and drought stress through improved water and nutrient transport, as well as regulation of stomatal activity^6^. These symbiotic associations represent an underexplored reservoir of bioactive metabolites, with *Aspergillus oryzae* serving as a prominent example.

Alzheimer’s disease (AD) is a progressive neurodegenerative disorder characterized by cognitive decline and driven by multifactorial pathologies, including acetylcholine depletion, extracellular *β*-amyloid (A*β*) plaque accumulation, and tau protein aggregation^7^. Two key enzymes are crucial to AD pathogenesis: acetylcholinesterase (AChE), which hydrolyses acetylcholine, and *β*-secretase (BACE-1), which catalyses the rate-limiting step in A*β* production^8^. Accordingly, inhibition of AChE and BACE-1 remains a cornerstone of current AD management and is also relevant to other therapeutic areas, including glaucoma, myasthenia gravis, and nerve injury^9,10^.

**Fig. 1:**
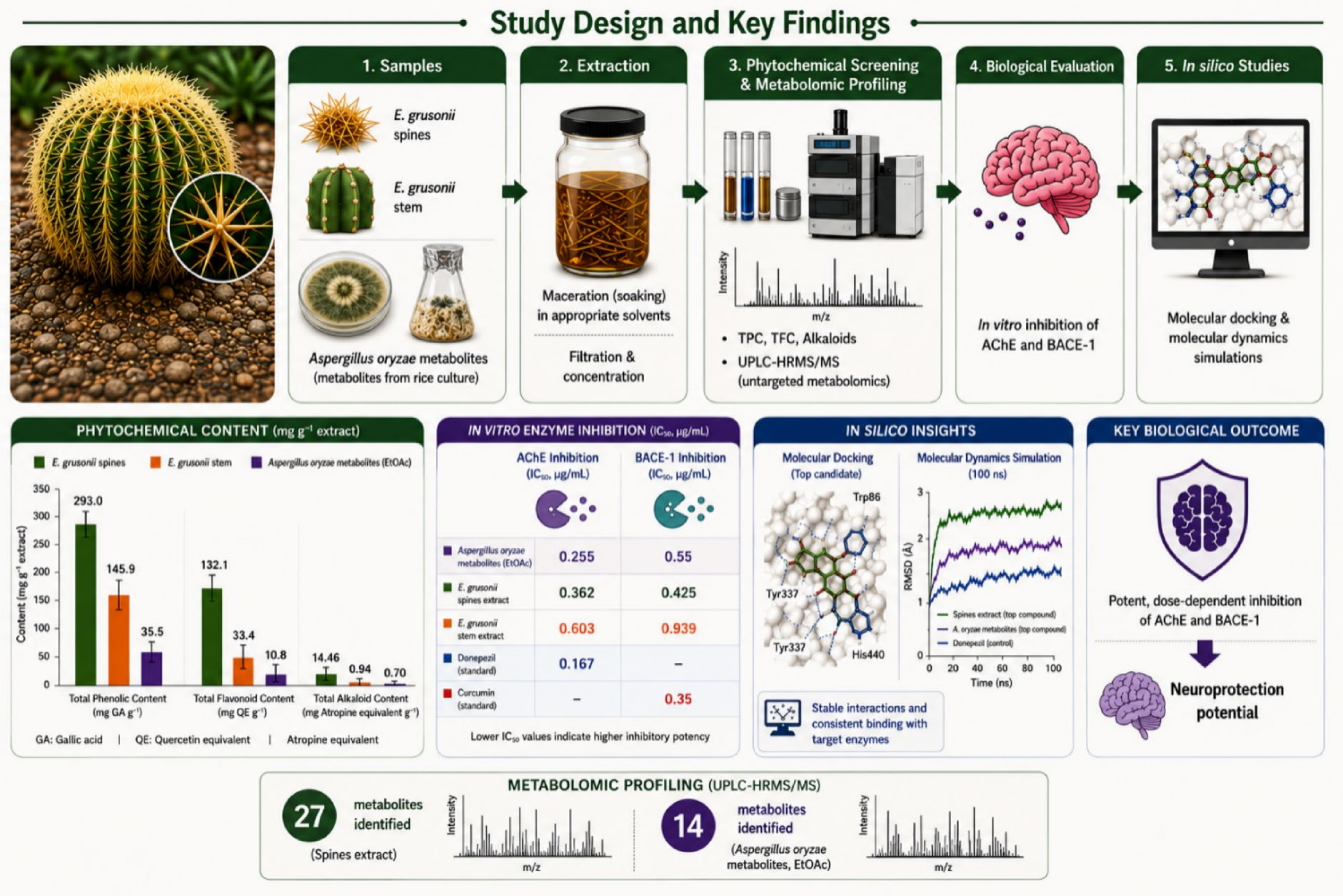
Schematic workflow of the current study.

Building upon the reported neuroprotective potential of *E. grusonii*^1^, the present work extends current knowledge through a comparative investigation of the chemical composition and neuroenzyme inhibition activities of *E. grusonii* spines and its associated endophytic fungus, *Aspergillus oryzae*. This integrated approach provides additional insights into the potential contribution of both plant and fungal metabolites to the observed biological activities. In the present study, all extracts were evaluated for their in vitro inhibitory activity against AChE and BACE-1, with total phenolic, flavonoid, and alkaloid contents quantified. The spines extractwas subjected to GC-MS analysis, while both the spines and *A. oryzae* extracts were profiled using UPLC-MS to identify key bioactive constituents. To elucidate potential mechanisms of action, molecular docking studies were performed to predict binding interactions with the active sites of AChE and BACE‑1. These predictions were subsequently validated through molecular dynamics (MD) simulation. A schematic workflow of the study is presented in Fig. 1.

## Materials and methods

### Plant material

Samples of *Echinocactus grusonii* Hildm. were collected in July 2023 from Helal cactus farm (30° 06’ 29.0” N, 31° 06’ 19.5” E). The species was authenticated by Prof. Abdo Marei Hamed, Professor of Plant Ecology in the Department of Botany and Microbiology, Faculty of Science, Al-Azhar University, Cairo, Egypt. Following collection, the spines were manually separated from the stems, air-dried, finely powdered, and then extracted separately using 80% methanol. The resulting crude extracts were concentrated under reduced pressure, yielding approximately 3 g of each sample.

### Isolation and purification of fungal material

The plant stems were washed with sterilized distilled water, treated with ethanol (70%) for 1–2 min, and ultimately air-dried under a laminar flow hood, the inner stem tissues were carefully dissected under sterile conditions and placed onto Sabouraud Dextrose Agar (SDA) plates containing antibiotics. After 3 days of incubation at room temperature, the hyphal tips of the fungi were removed and transferred to a fresh SDA medium. A pure strain was isolated by repeated inoculation. The isolated pure fungal strain was identified as *Aspergillus oryzae* by Dr. Amal A. E. Mekawy, Regional Center for Mycology and Biotechnology (RCMB), Al-Azhar University.

### Extraction and fractionation of fungal metabolites

*Aspergillus oryzae* fungus mass growth was performed on a solid culture medium containing 100 g basmati rice. The cultures were then incubated at room temperature without shaking for 21 days. For extraction of fungal metabolites, the ethyl acetate (EtOAc, 250 mL x3) was added to the fungal culture and left for 48 h to ensure complete extraction. The remaining culture media was extracted with 100% methanol (MeOH). Following concentration under reduced pressure, the obtained extracts were *Aspergillus oryzae* EtOAc (Ao-E, 5.8 g), *Aspergillus oryzae* MeOH (Ao-M, 3.0 g).

### Chemicals and reagents

All chemicals and reagents used in this study were of analytical or reagent grade. Solvents, including MeOH and EtOAc, were used for the extraction of plant and fungal materials. Reference standards were purchased from Sigma-Aldrich (St. Louis, MO, USA).

### In vitro acetyl cholinesterase (AChE) and *β*-secretase (BACE-1) inhibition assays

The AChE and BACE-1 inhibitory assays were performed using AChE inhibitor screening assay kit USA (K197-100) and BACE-1 inhibitor screening assay kit, Bio Vision, USA (K720-100), following the method previously mentioned in literature^1^.

### Analysis of total phenolic content

Total phenolic content was determined as stated in Folin–Ciocalteau method with some modifications^11^. Three independent samples of each extract (500 and 1000 µg/mL) were used. Gallic acid calibration solutions of 15.71–300 µg/mL concentrations were prepared in duplicates. Then, 1 mL of each extract was dissolved in 2 ml of MeOH, 500 µL of each extract was mixed with 2.5 mL Folin–Ciocalteau reagent (Merck, Darmstadt, Germany), diluted ten-fold, and 2.5 mL (75 g/L) Na_2_CO_3_. After incubation at 25 °C for 2 h, the absorbance was measured at 765 nm using a BioTek Synergy HTX Multi-Mode Reader. Total phenolic content was expressed as milligrams of gallic acid equivalent (GAE) per gm of dry extract^11^.

### Analysis of total flavonoid content

Total flavonoids were determined by a modified AlCl_3_ colorimetric method. 1 mL of each extract was dissolved in 2 mL of MeOH in a 10 mL volumetric flask. Then, 5% NaNO_3_, 5% NaOH, and 7% AlCl_3_ solutions were prepared in a 25 ml of volumetric flask. Following that, 200 µL of each extract was mixed with 75 µL of 5% NaNO_3_ in a sealed glass vial and left for 5 min at room temperature. Moreover, 1.25 mL of AlCl_3_ and 0.5 mL NaOH were added to each vial, and the mixture was sonicated and incubated for 5 min at room temperature. After incubation, the absorbance was measured at 510 nm. The flavonoids content of the extracts were estimated by using quercetin standard calibration curve and the obtained results were expressed as microgram of quercetin equivalent (QE) per 1 g of dry extract^11,12^.

### Analysis of total alkaloid content

In this study, total alkaloids were estimated as previously described in the literature^13^. Briefly, bromocresol green (BCG) solution was prepared by heating 69.8 mg bromocresol green, 3 mL 2 N NaOH, and 5 mL distilled water till completely dissolved. The solution was then diluted to 1000 mL with distilled water. Phosphate buffer solution (PH 4.7) was prepared by adjusting PH of sodium phosphate 2 M (71.6 g Na_2_HPO_4_ in 1000 mL distilled water) to 4.7 with citric acid (42.02 g citric acid in 1000 mL). The residue of each extract was dissolved in 2 N HCl and filtered. in a separating funnel, 1 mL of solution was washed with 10 mL chloroform (3 times). Then, the pH of the solution was rendered neutral with 0.1 N NaOH. Following that, 5 mL of BCG solution and 5 mL Phosphate buffer were added to the solution and the mixture extracted with 5 mL chloroform by vigorous shaking. The collected extracts were diluted with chloroform, and the absorbance was measured at 470 nm.

Additionally, the standard solution of atropine was made by dissolving 10 mg of a pure atropine (Sigma Chemical, USA) in 10 mL distilled water and aliquots of 5, 10, 20, 40 and 60 ug/mL transferred to different separating funnels. Then, 5 mL of BCG solution and 5 mL Phosphate buffer were added, followed by extraction with chloroform and measure the absorbance at 470 nm.

### UPLC-ESI-MS/MS analysis

Chemical profiling of *Echinocactus grusonii* spines extract, in addition to the EtOAc extract of the isolated endophytic fungus *Aspergillus oryzae* was done utilizing ESI- MS/MS coupled to UPLC as previously reported by Talaat et al.^14^.

### Molecular docking and molecular dynamics simulations (MD)

See supplementary information.

### Statistical analysis

Statistical analysis was carried out using Graph Pad Prism version 8.0.2. ANOVA with Tukey’s multiple comparison test, with the level of significance *p* < 0.05, was conducted to compare the mean values of the results. All experiments were performed using three independent biological replicates (*n* = 3), and the results obtained were expressed as mean ± SD.

## Results and discussion

### Total phenolics, flavonoids, and alkaloids in *E. grusonii*

Plants produce phenolic compounds (polyphenols) and flavonoids as key adaptive strategy to combat oxidative damage caused by abiotic stresses such as drought, UV radiation, and salinity, as well as biotic threats including herbivores and pathogens. Additionally, alkaloids, another class of nitrogenous secondary metabolites, also play a vital role in plant defence against environmental stressors^15,16^. The total phenolic content (TPC) of extracts from *E. grusonii* (stems and spines) and its endophytic fungus *Aspergillus oryzae* was determined using the Folin–Ciocalteau assay. This method relies on an electron transfer from phenolic compounds to a phosphomolybdic/phosphotungstic acid complex, producing a blue color detectable at 760 nm. The intensity correlates with reducing power and is measured as gallic acid equivalents (GAE). Using a gallic acid calibration curve ($\:\mathrm{y}=528.44\mathrm{x}-42.318$, $\:{\mathrm{R}}^{2}=0.998$), the spines extract showed the highest TPC (293.0 ± 3.2 mg GAE/g), followed by the stems (145.9 ± 2.1 mg GAE/g), as seen in Table 1. The study also quantified total flavonoid content (TFC) and alkaloid content. The spines extract again exhibited the highest levels for both, with a TFC of 132.1 ± 1.3 mg QE/g and an alkaloid content of 14.46 ± 0.39 mg atropine equivalents/g, as detailed in Table 1.

**Table 1:** Total phenolic content (TPC) [mg GA g^− 1^], total flavonoid content (TFC) [mg QE g^− 1^] and alkaloid content [mg Atropine g^− 1^] in different extracts of EG. The results are expressed as mean ± SD (*n* = 3).

Sample	Total Phenolic Content [mg GA g^− 1^]	Total Flavonoid Content [mg QE g^− 1^]	Total Alkaloid content [mg Atropine equivalent g^− 1^]
*E. grusonii* spines	293.0 ± 3.2	132.1 ± 1.3	14.46 ± 0.39
*E. grusonii* stem	145.9 ± 2.1	33.4 ± 0.6	0.94 ± 0.09
*Aspergillus oryzae* EtOAc	35.5 ± 0.8	10.8 ± 0.3	0.70 ± 0.07

### Determination of individual compounds by gas chromatography-mass spectrometry (GC-MS) analysis

 Gas chromatography–mass spectrometry (GC-MS) analysis of the *E. grusonii* spines extract revealed 11 compounds, as detailed in Table S1. The main compounds detected were glycerol 1-palmitate (19.96%), and 2,3-dihydroxypropyl stearate (15.33%). Additionally, three alkaloids were detected in the extract named, 3-oxo-20-methy l-11-*α*-hydroxy conanine-1,4-diene, 3*α*-hydroxy-6-benzyl-6-azacholest-4-en-7-one, and 2-acetyl-3-(2-cinnamido) ethyl-7-methoxyindole. The identification was made using the mass spectral data as well as the published data and NIST Mass Spectral Library (December 2005).

### Profiling of *Echinocactus grusonii* metabolites by UPLC- ESI- MS/MS analysis

#### Annotation of metabolites in *Echinocactus grusonii* spines extract

**Fig. 2:**
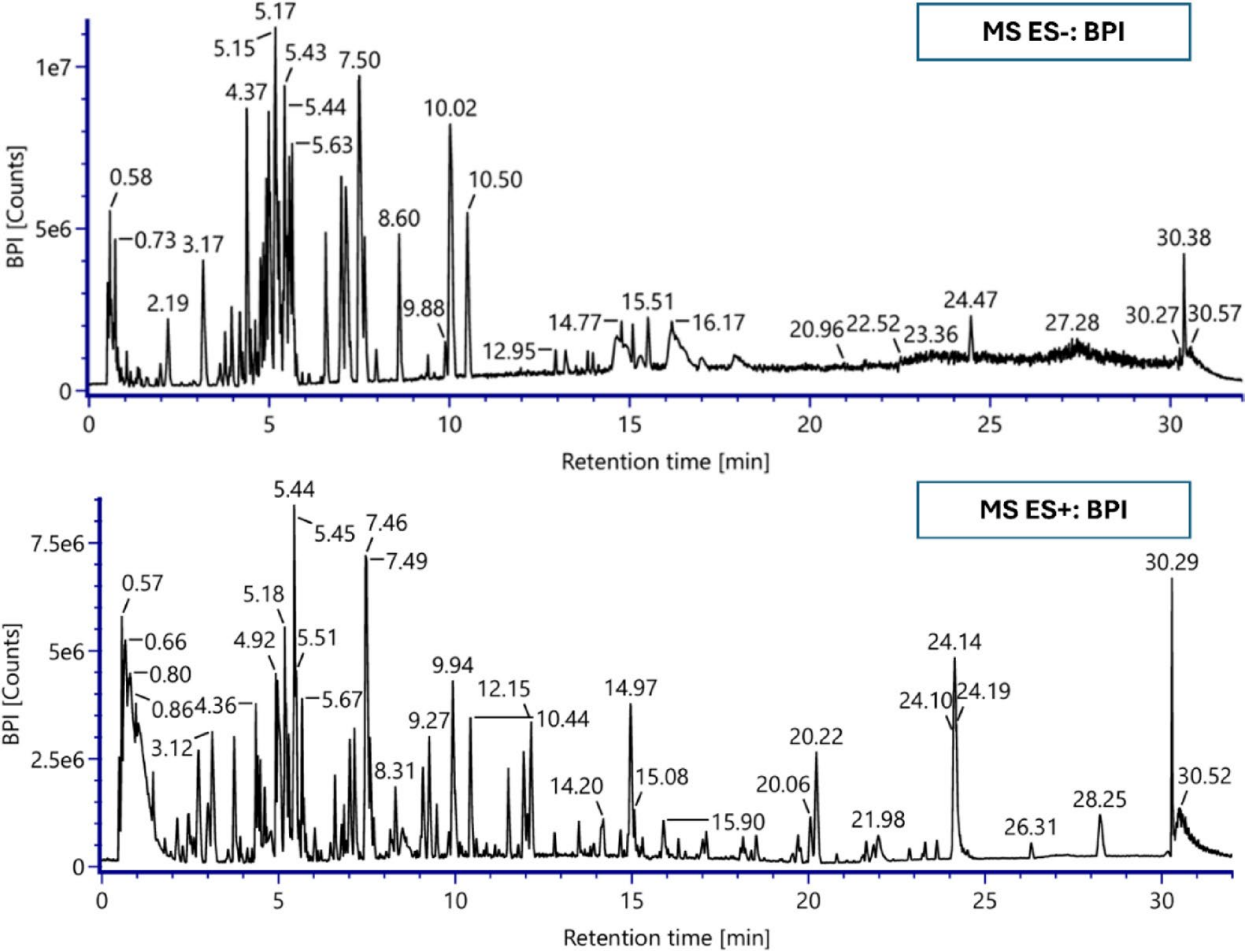
Base peak ion (BPI) chromatograms of *Echinocactus grusonii* spines extract in positive and negative ionization modes.

**Fig. 3:**
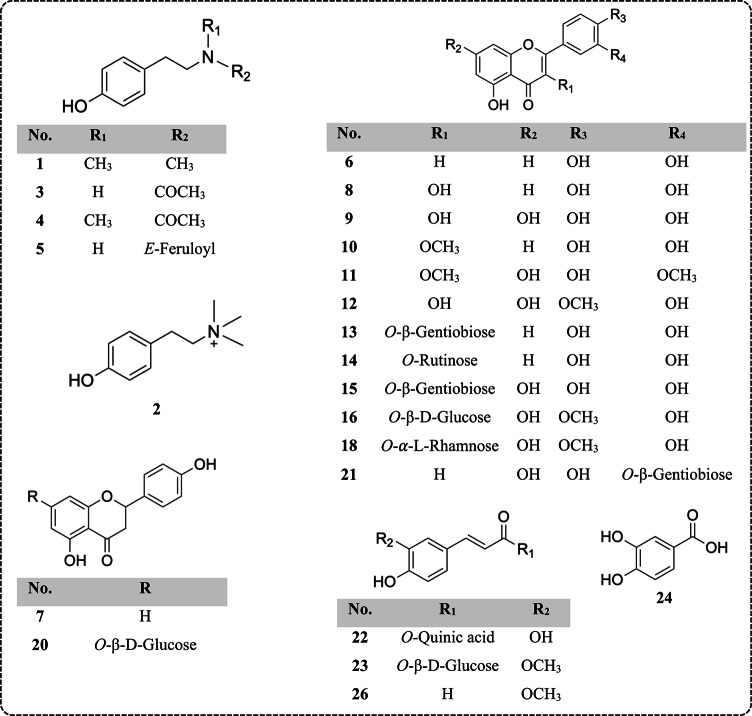
Representative compounds identified in *Echinocactus grusonii* spines extract.

 The methanolic extract of *Echinocactus grusonii* spines was analysed by ultra-high-performance liquid chromatography-tandem mass spectrometry (UPLC-MS/MS) to characterize its principal bioactive constituents. The resulting base peak ion (BPI) chromatograms in both positive and negative electrospray ionization modes are presented in Fig. 2. UPLC-MS/MS analysis of the spines extract in both positive and negative ESI modes enabled the tentative identification of 27 metabolites. These compounds, which include alkaloids, phenolic acids, and flavonoids, were annotated based on their retention times, observed MS/MS fragment ions, and fragmentation patterns by comparison with literature data (Table S2). Representative compounds identified are illustrated in Fig. 3.

##### Alkaloids

The presence of alkaloids is a distinctive chemotaxonomic characteristic of cacti species^17^, with phenylethylamines and tetrahydroisoquinolines representing two major classes^18^. These alkaloids are particularly prevalent in the Cactoideae subfamily, revealing their role as chemotaxonomic markers. Supporting this, our previous study on *Echinocactus grusonii* stems extract led to the isolation of two rare chlorinated phenylethylamine alkaloids^1^, further confirming the presence of this alkaloid class within the species. To investigate the alkaloid constituents of *Echinocactus grusonii* spines extract, UPLC-MS/MS analysis was performed. This led to the tentative identification of five alkaloids.

Compounds **1–5** were characterized as phenethylamine derivatives. Compound **1** was tentatively identified as hordenine. It displayed a precursor ion at *m/z* 166.1233, with characteristic fragment ions at *m/z* 121 and 103^19^. Similarly, compound **2** was identified as candicine, a quaternary ammonium alkaloid, characterized by a parent ion at *m/z* 180.1383 [M + H]^+^ and a daughter ion at *m/z* 121 resulting from from the loss of a trimethylamine moiety *N*(CH_3_)_3_^20^. Compound **3** was recognized as *N*-acetyl tyramine indicated by a protonated molecular ion [M + H] ^+^ at *m/z* 180.1026. A key fragment ion at *m/z* 166 corresponds to the loss of methyl group. Besides, the break of the C-N bond between *N*-CO-CH_3_ and CH_2_ resulted in fragment ions appeared at *m/z* 121^21^. Compound **4**, *N*-methyl-*N*-(4-hydroxyphenethyl) acetamide, is *N*-methylated derivative of **3** identified by a precursor ion at *m/z* 194.1172, with characteristic fragments at *m/z* 180 and 165 denoted consequent demethylations of acetyl and *N*-CH_3_ groups^21^. Finally, **5** was characterized as *N*-*trans* feruloyl tyramine, as it exhibited a protonated base peak at *m/z* 314.1371. Characteristic fragments were observed at *m/z* 177 and 121, corresponding to the cleavage of the feruloyl amide and subsequent feruloyl moiety, respectively^22^.

##### Flavonoids

Flavonoids, present as both aglycones and glycosides, are widely distributed secondary metabolites in Cactaceae species^2^. In xerophytic plants such as *Echinocactus grusonii*, flavonoids play essential roles in adaptation to harsh environments by regulating cell differentiation and growth, and by providing protection against biotic and abiotic stresses^23^. In the current study, UPLC-MS/MS analysis enabled the tentative identification of several flavonoids (**6**–**21)** within the *E. grusonii* spines extract as seen Table S2.

Compound **6** (*m/z* 271.0600 [M + H]⁺) was characterized as apigenin, showing a fragment at *m/z* 153 ([¹,³A]⁻, RDA cleavage)^24^. Compounds **7** and **20** corresponded to naringenin (*m/z* 273.0758 [M + H]⁺) and naringenin-7-*O*-glucoside (*m/z* 433.1150 [M–H]⁻)^25–28^. Compounds **8**,** 10**, **13**, and **14** were identified kaempferol (*m/z* 287.0551 [M + H]⁺), isokaempferide (*m/z* 301.0711 [M + H]⁺), kaempferol-3-gentiobioside (*m/z* 609.1467 [M–H]⁻), and kaempferol-3-*O*-rutinoside (*m/z* 595.1663 [M + H]⁺and *m/z* 617.1485 [M + Na]^+^), respectively^29–31^. Moreover, compounds **9**, **11**, **12**, and **15** were annotated quercetin (*m/z* 303 [M + H]⁺), quercetin dimethyl ether (*m/z* 331.0816 [M + H]⁺), isorhamnetin (*m/z* 317.0658 [M + H]⁺), and quercetin-3-gentiobioside (*m/z* 625.1418 [M–H]⁻), respectively^31–36^. Compounds **16–19** were determined as isorhamnetin-3-rhamnoside, isorhamnetin-3-galactoside, isorhamnetin 3-*O*-rhamnoside, and isorhamnetin-3-*O*-(2-hexosyl) hexoside, respectively. Finally, compound **21** was suggested as luteolin-7-*O*-gentiobioside (*m/z* 611.1613 [M + H]⁺)^37–40^.

##### Phenolic acids

Three phenolic acids were tentatively identified from the methanolic extract of *E. grusonii* spines, including chlorogenic acid (**22)** (*m/z* 353.0888 [M–H]⁻), ferulic acid *β*-glucoside (**23**) (*m/z* 355.1045 [M–H]⁻), and protocatechuic acid (**24**) (*m/z* 153.0195 [M–H]⁻)^41,42^.

##### Other miscellaneous compounds

Compound **25** was identified as sucrose with *m/z* 365.1058 [M + Na] ⁺. Compound **26** was determined to be ferulaldehyde (C₁₀H₁₀O₃) at *m/z* 179 [M + H] ⁺. Finally, stigmasterol (**27**) was annotated from a pseudo-molecular ion peak at *m/z* 413.2653 [M + H]⁺^43–46^.

#### Annotation of metabolites in *Aspergillus oryza* isolated from *Echinocactus grusonii*

**Fig. 4:**
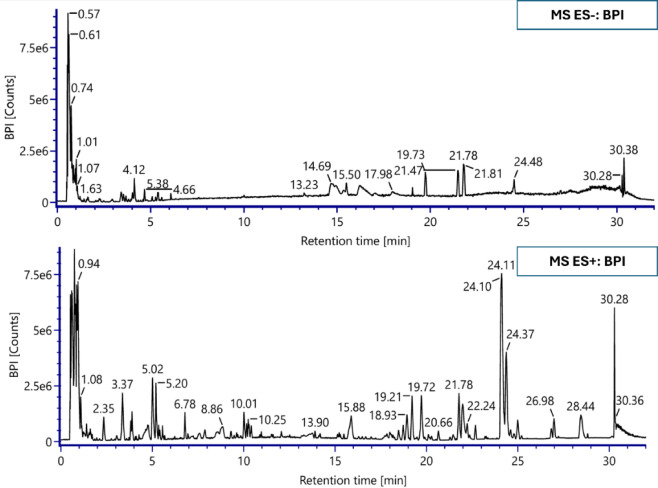
Base peak ion (BPI) chromatograms of *Aspergillus oryza* EtOAc extract in positive and negative ionization modes.

**Fig. 5:**
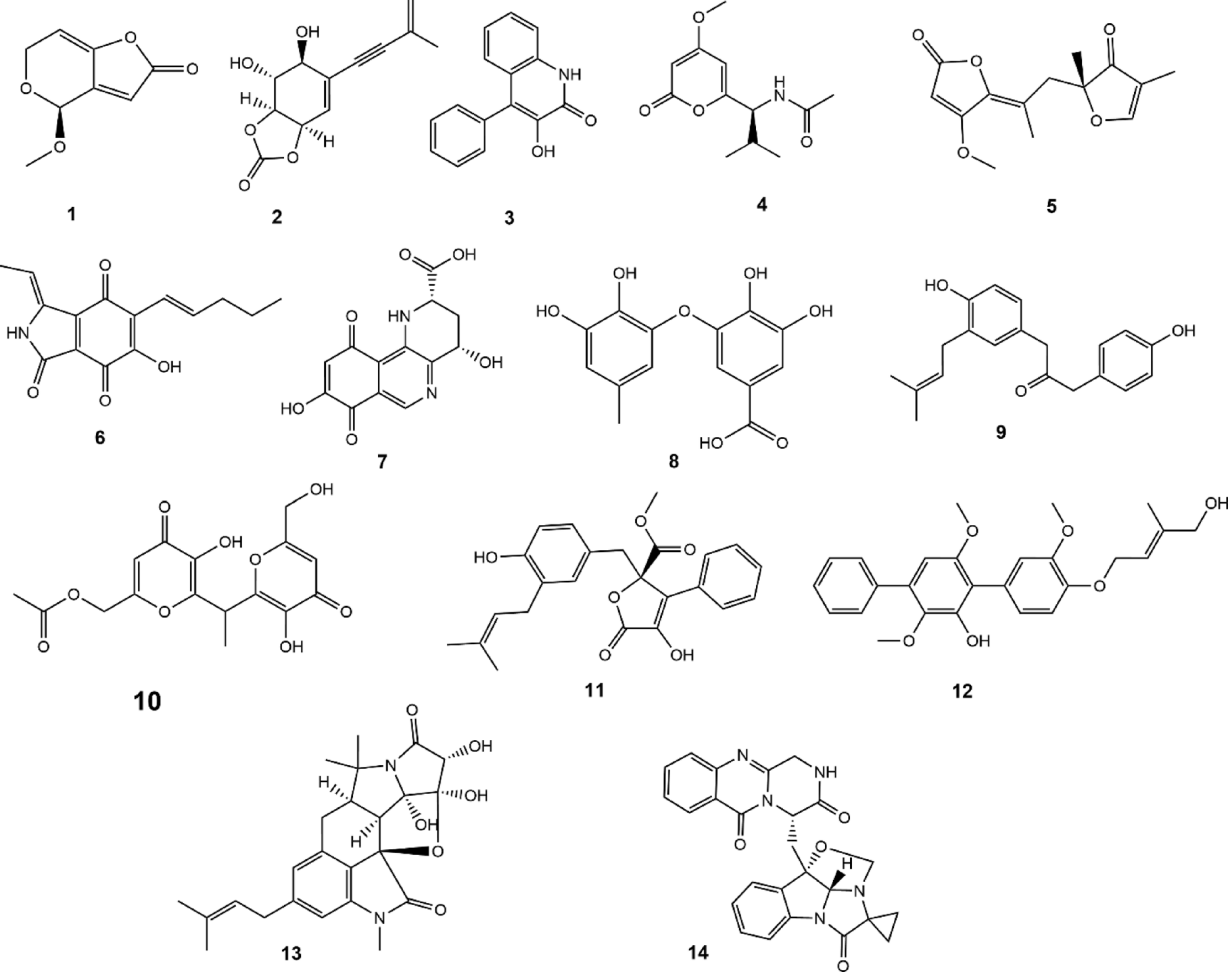
Identified compounds from *Aspergillus oryzae* EtOAc extract.

 The ethyl acetate (EtOAc) extract derived from *Aspergillus oryzae* isolated from *E. grusonii* stems was analysed by UPLC-MS/MS to identify its major active constituents. Base peak ion (BPI) chromatograms were acquired in both positive and negative ionization modes (Fig. 4). By comparing the obtained LC-MS data with previously reported metabolites from *Aspergillus* species, 14 compounds were tentatively identified. These included polyketides, alkaloids, terpenoids, pyrones, phenolics, and other classes of secondary metabolites (Table S3, Fig. 5).

Aspergilsmin C (**1**) is a polyketide previously isolated from *A. giganteus* and reported to exhibit anticancer activity, including significant anti-angiogenic functions^47^. Aspergillusol B (**2**) is a cyclic carbonate reported from the soil fungus *Aspergillus* sp. PSU-RSPG185^48^. Viridicatin (**3**), featuring a 2-quinolone/quinazoline core, is a phenyl quinolone previously reported from *A. carneus*^49^. Fumisoquin C (**7**) is an isoquinoline alkaloid reported from *A. fumigatus*^50^, while versiquinazoline F (**14**) is a fumiquinazoline alkaloid previously reported from *Aspergillus versicolor*. Although the anti-Alzheimer potential of these alkaloids has not been examined, prior studies on structurally related isoquinolines have demonstrated AChE inhibitory activity and neuroprotective effects^51^. Campyrone C (**4**) is an *α*-pyrone derivative isolated from *A. niger*^52^, while aspersclerotiorone A (**5**) is a γ-butenolide-furanone dimer reported from *A. sclerotiorum*^53^. Pyranterrone A (**6**), an isoindole, was isolated from *A. terreus*^54^. Aspergilol F (**8**) is an antioxidant phenolic compound reported from *A. versicolor*^55^. Terrusnolide A (**9**) is an anti-inflammatory butenolide obtained from an endophytic *Aspergillus* sp. isolated from *Tripterygium wilfordii*^56^. Dikojiacid A (**10**) is kojic acid derivative from *A. flavus* GZWMJZ-288^57^. Versicolactone B (**11**) is a butyrolactone isolated from *A. versicolor* with reported antiviral activity^58^. Prenylterphenyllin F (**12)** is a prenylated *p*-terphenyl from *A.candidus* LDJ-5; this class of compounds has demonstrated *α*-glucosidase inhibitory, cytotoxic, and antibacterial activities^59^.

### In vitro acetylcholinesterase (AChE) and *β*-secretase (BACE-1) inhibition activity

AChE and BACE-1 are recognized therapeutic targets in neurodegenerative disease research, and their inhibition represents a promising strategy not only for the management of Alzheimer’s disease but also for other neurological and cognitive disorders^1,9,10^ Accordingly, four extracts including *Aspergillus oryzae* EtOAc (Ao-E), *Aspergillus oryzae* MeOH (Ao-M), *E. grusonii* stems, and *E. grusonii* spines, were evaluated for their in vitro inhibition of AChE and BACE-1. As summarized in Table 2, all extracts exhibited dose-dependent inhibition of both enzymes, with the spines and Ao-E extracts demonstrating the most potent activity. The spines extract recorded IC₅₀ values of 0.362 ± 0.012 µg/mL against AChE and 0.425 ± 0.014 µg/mL against BACE-1. Similarly, the Ao-E extract showed IC₅₀ values of 0.255 ± 0.008 µg/mL against AChE and 0.550 ± 0.018 µg/mL against BACE-1. The pronounced bioactivity observed is most likely attributed to the enriched content of secondary metabolites within these crude extracts.

**Table 2:** Acetylcholinesterase (AChE) and *β*-secretase (BACE-1) inhibitory activity of *E. grusonii* and *Aspergillus oryzae* extracts. All determinations were carried out using three independent biological replicates (*n* = 3), and values are expressed in µg/mL concentration ± SD.

Compound	AChE inhibition (IC₅₀, µg/mL)	BACE-1 inhibition (IC₅₀, µg/mL)
*Aspergillus oryzae* EtOAc	0.255 ± 0.008	0.55 ± 0.018
*Aspergillus oryzae* MeOH	3.18 ± 0.104	1.882 ± 0.062
Spines extract	0.362 ± 0.012	0.425 ± 0.014
Stem extract	0.603 ± 0.019	0.939 ± 0.031
Donepezil	0.167 ± 0.006	–
Curcumin	–	0.35 ± 0.012

It is important to compare the neuroprotective activity observed in our study with previously reported natural inhibitors from related cactus species. Literature survey indicated that extracts of *Opuntia microdasys* var. rufida have demonstrated potent butyrylcholinesterase inhibition, with IC₅₀ values as low as 0.04 mg/mL for the ethyl acetate flower extract and 0.08 mg/mL for the methanolic fruit extract of *O. leptocaulis.* Furthermore, flavonol glycosides isolated from *O. microdasys* (e.g., isorhamnetin and quercetin derivatives) were shown through docking studies to interact favorably with cholinesterase active sites, supporting their role as specific inhibitors^60^. In addition, previous studies reported that hordenine possesses neuroprotective properties against AlCl₃-induced Alzheimer’s disease^61^, while chlorinated derivatives of hordenine exhibited strong inhibitory activity against both AChE and BACE-1^1^. Moreover, apigenin has been shown to enhance cholinergic function through multiple mechanisms, including elevating acetylcholine levels in the brain, improving cholinergic transmission, and protecting the structural integrity of the blood–brain barrier. It also counteracts amyloid-*β* effects by restoring acetylcholine secretion and promoting hippocampal choline uptake. In animal models, apigenin alleviates memory impairment through acetylcholinesterase inhibition and increased cortical acetylcholine, thereby improving learning and memory performance^62–64^. Additionally, quercetin demonstrated neuroprotective effects in an AlCl₃-induced Alzheimer’s rat model by improving memory deficits and modulating key AD-related genes, including reductions in APP, BACE-1, and presenilin-1 (PSEN1)^65^. In addition to these effects, the AChE inhibitory activity of quercetin and its glycosides has been extensively reported^66^. Naringenin, kaempferol, and isokaempferide displayed both in vitro and in silico inhibitory activity against AChE and BACE-1^1^. The neuroprotective properties of naringenin have been attributed to multiple mechanisms, including the reduction of neurotoxicity, oxidative stress, and neuroinflammation, as well as its anti-amyloidogenic potential^67^. Similarly, kaempferol and its derivatives exert neuroprotective effects by targeting key pathological pathways, such as inhibiting protein aggregation, reducing inflammation, and mitigating oxidative stress^68^. Furthermore, isorhamnetin demonstrated significant neuroprotective effects in STZ-induced diabetic rats^69^. Previous studies have demonstrated the ability of chlorogenic acid to inhibit acetylcholinesterase ex *vivo* in the hippocampus and frontal cortex of mice, as well as in in vitro assays. Molecular docking simulations further suggested a potential binding interaction of chlorogenic acid with the AChE enzyme, reinforcing its role as a promising natural inhibitor^70,71^.

### Molecular docking study

**Fig. 6:**
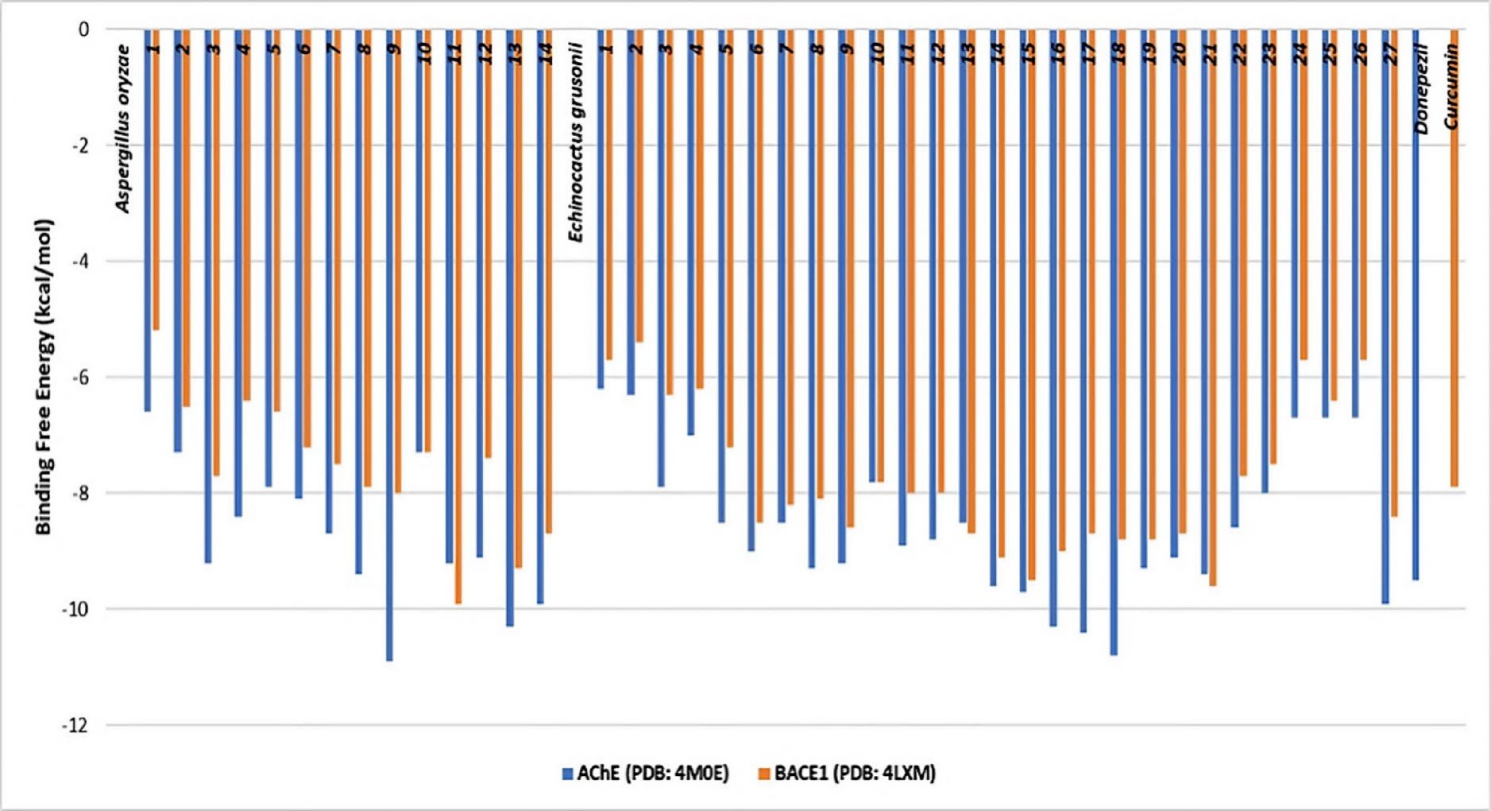
Binding free energies of EtOAc *Aspergillus oryzae* and MeOH *Echinocactus grusonii* spines constituents toward AChE (PDB: 4M0E) and BACE-1 (PDB: 4LXM).

Molecular docking was employed to investigate the binding interactions between compounds identified in *A. oryzae* and *E. grusonii* spines extracts with AChE (PDB: 4M0E) and BACE-1 (PDB: 4LXM). In agreement with the in vitro results, the highest docking score against 4M0E was identified among the constituents of *A. oryzae* EtOAc extract for compound **9** (terrusnolide A), −10.9 kcal/mol), as shown in Fig. 6. Other compounds in both extracts may be considered potent, with higher scores, such as aspergilline C (**13**) in the EtOAc *A. oryzae* extract (−10.3 kcal/mol) and isorhamnetin-3-*O*-glucoside (**16**), isorhamnetin-3-galactoside (**17**) and isorhamnetin 3-*O*-rhamnoside (**18**) in the MeOH *E. grusonii* spines extract (−10.3 to −10.8 kcal/mol). On the other hand, compounds **11** (versicolactone B) in EtOAc *A. oryzae* and **15**(quercetin-3-gentiobioside) in MeOH *E. grusonii* spines extracts showed close binding affinities toward 4LXM (−9.9 and − 9.6 kcal/mol). Again, aspergilline C (**13**) from EtOAc *A. oryzae* extract, in addition to isorhamnetin-3-*O*-glucoside (**14**), isorhamnetin 3-*O*-rhamnoside (**16**), and luteolin-7-*O*-gentiobioside (**21**) of MeOH *E. grusonii* spines extract, recorded higher binding free energies to 4LXM, ranging from − 9 to −9.5 kcal/mol. Interestingly, the above constituents showed higher scores than positive standards used, such as donepezil (−9.5 kcal/mol) for AChE and curcumin (−7.9 kcal/mol) for BACE-1. The most potent constituents in EtOAc *A. oryzae* extract (**9**, **11**, and **13**) were members of butenolide, butyrolactone, and alkaloid classes. These computational findings are consistent with the observed in vitro enzyme inhibition.

**Fig. 7:**
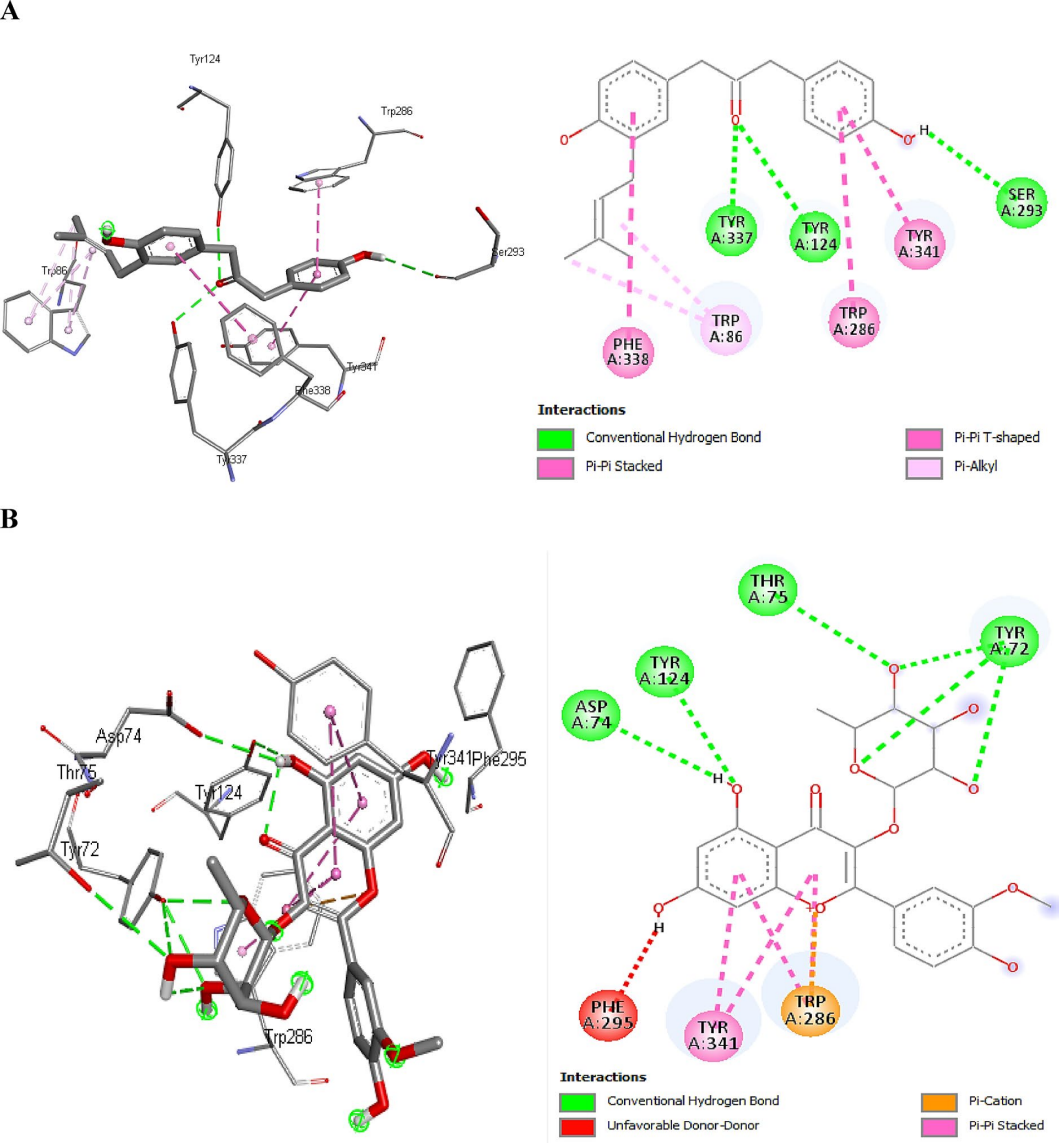

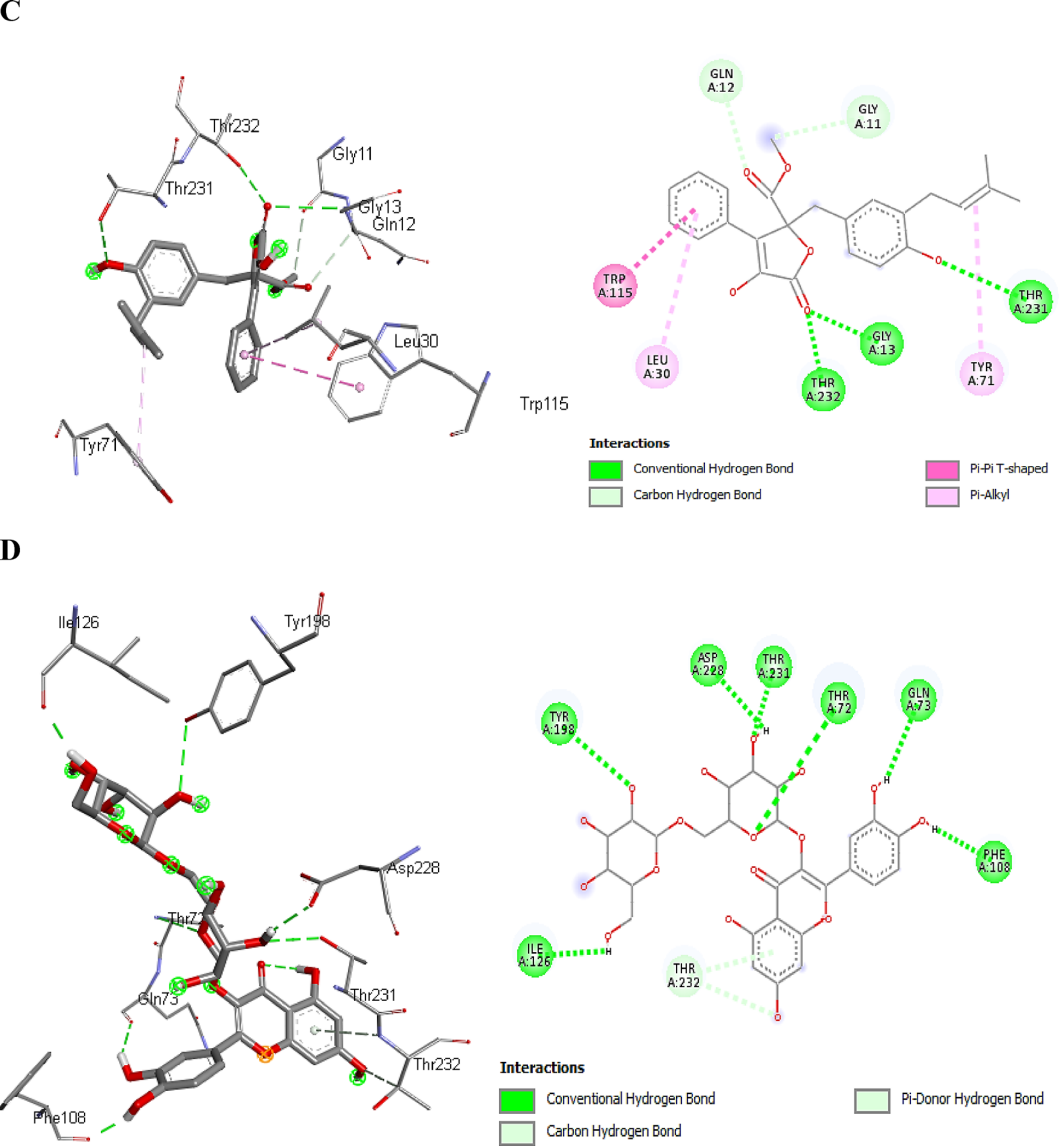
Docking interactions of potent constituents in EtOAc *A. oryzae* compounds **9** (**A**) and **11** (**C**) and MeOH *E. grusonii* spines compounds **18** (**B**) and **15** (**D**) against AChE (PDB: 4M0E) and BACE-1 (PDB: 4LXM).

Based on these findings, it was necessary to characterize the types of interactions formed by the potent constituents—EtOAc *A. oryzae t*errusnolide A (**9**) and versicolactone B (**11**), as well as MeOH *E. grusonii* spines isorhamnetin-3-*O*-rhamnoside (**18**) and quercetin-3-gentiobioside (**15**)—against AChE (PDB: 4M0E) and BACE-1 (PDB: 4LXM), as shown in Fig. 7A–D. In agreement with Ferreira De Freitas and Schapira (2017)^72^, hydrogen bonds, hydrophobic contacts, and π-stacking were the dominant, followed by weak hydrogen bonds and cation–π interactions. Conventional hydrogen bonds are mainly responsible for the higher docking scores in the investigated complexes. O–H⋯O interactions were observed in 4M0E-**9** and **18** of EtOAc *A. oryzae* and MeOH *E. grusonii* spines between the hydroxyl group of TYR A:124, TYR A:337, TYR A:72, THR 75, **9**, and **18**, the carbonyl, hydroxyl, and carboxylate groups of the ligands, SER A:293, and ASP A:74 (Fig. 7A and B). The same type of bond shown between THR A:231, THR A:232, TYR A:198, and THR A:231 hydroxyl groups of 4LXM and the carbonyl as well as the hydroxy groups of compounds **11** and **15** of EtOAc *A. oryzae* and MeOH *E. grusonii* spines. Interestingly, the hydroxyl groups of **15** made 5 bonds with the carbonyl of GLN A:73, PHE A:108, ILE A:126, ASP A:228, and the ligand itself. On the other hand, *N*–H⋯*O* interactions were detected between GLY A:13 and THR A:72 of 4LXM and the carbonyl and *O*-pyran of the constituents **11** and **15** in EtOAc *A. oryzae* and MeOH *E. grusonii* spines (Fig. 7C and D). According to Chen and Kurgan (2009)^73^, who investigated hydrogen bonds between amino acids in proteins and small ligands, hydrophilic residues such as TYR, THR, and GLY were more involved in this type of bonding, acting as donors more than acceptors, which is consistent with our findings.

The cation–π interaction observed between the positive pyran ring of **18** and the π-orbitals of TRP A:286 (Fig. 7B) is a potent, general non-covalent binding force observed across a wide range of biological contexts, including interactions between TRP residues and ACh^74^. π-π stacked and T-shaped interactions are two common, non-covalent aromatic-aromatic orientations, with stacked featuring parallel ring faces, while T-shaped involves the edge of one ring facing the centre of another. According to Wilson and colleagues^75^, and in agreement with our findings, stacked interactions are more abundant, while T-shaped interactions are often driven by electrostatic attraction between edge hydrogens and the ring centre. As shown in Fig. 7A and B, π-π stacked bonds between TRP A:286 and TYR A:341 residues of 4M0E and the compounds **9** and **18** of EtOAc *A. oryzae* and MeOH *E. grusonii* spines, while π-πT-shaped bonds were observed between PHE A:338 of 4M0E and TRP A:115 of 4LXM and **9** and **11** of EtOAc *A. oryzae.* Almost 50% of all π-stacking interactions are observed between the aromatic ring of phenylalanine, tyrosine (36.8%), tryptophan (8.7%), and histidine (5.1%) with that of the ligand^72^. Hydrophobic pi-alkyl bonding was found only in **9** and **11** of EtOAc *A. oryzae* with the pi-orbitals of TRP A:86 and TYR A:71 of 4M0E and 4LXM. Finally, carbon as a proton donor in GLN A:12 and THR A:232 of 4LXM was bonded to **11** and **15** of the EtOAc *A. oryzae* and MeOH *E. grusonii* spines, respectively, as proton acceptors.

### Molecular dynamics (MD) simulation

**Fig. 8:**
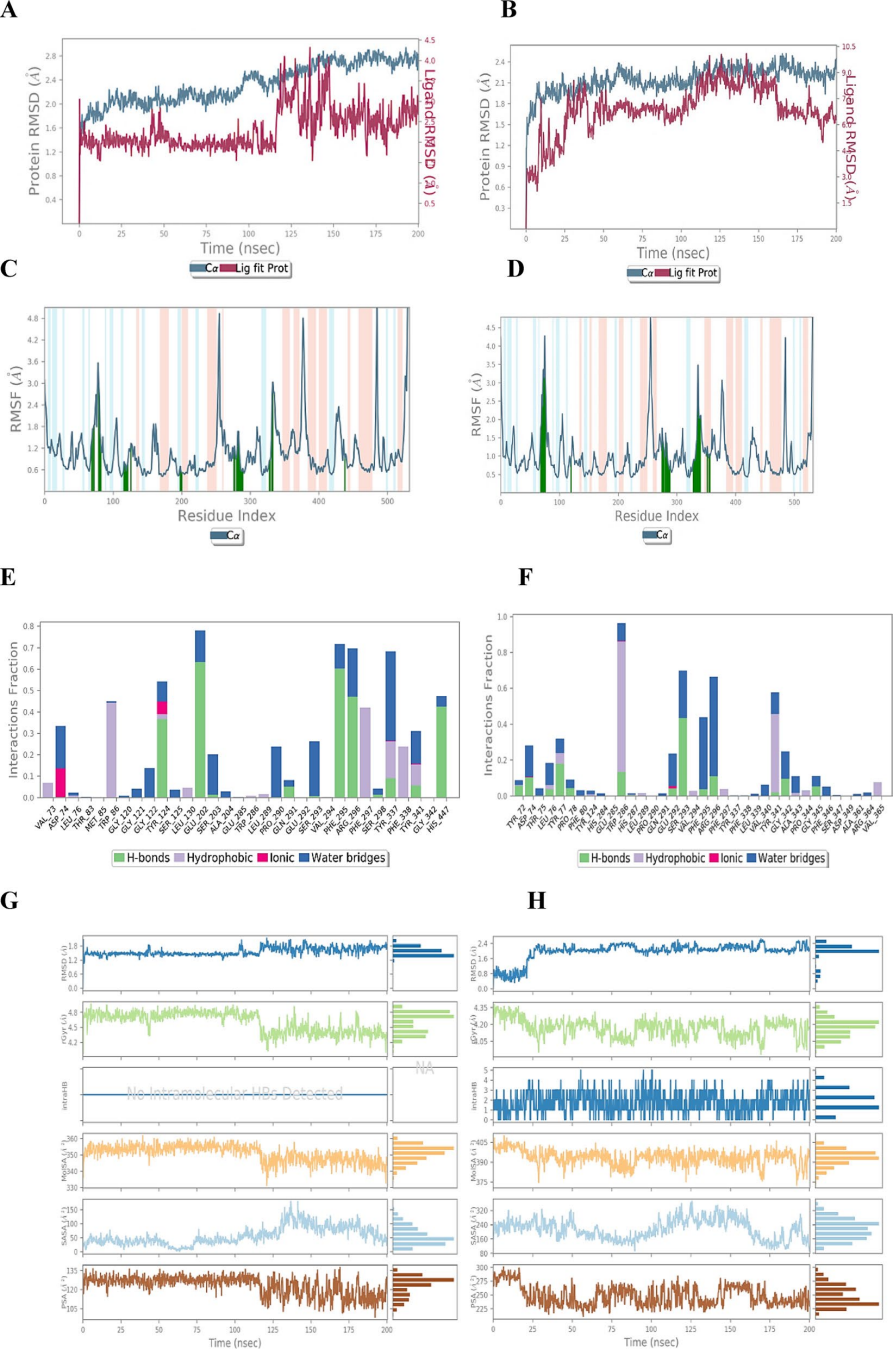
MD simulations for the interactions of EtOAc *A. oryzae* compound (**9)** (**A**, **C**, **E**, and **G**) and MeOH *E. grusonii* spines compound (**18)** (**B**, **D**, **F**, and **H**) against AChE (PDB: 4M0E).

It was necessary to study the MD simulation interactions of the potent metabolites with AChE and BACE-1 targets, in line with the study’s aim. Figure 8 illustrates and compares the MD simulations of the potent components terrusnolide A (**9**) of EtOAc *A. oryzae* and Isorhamnetin 3-*O*-rhamnoside (**18**) of MeOH *E. grusonii* spines, against AChE (PDB: 4M0E). The Root Mean Square Deviation (RMSD) of the enzyme (C*α*) indicates a stable protein backbone with values around 1.5–2.0 Å. AChE does not undergo major conformational changes in either complex (Fig. 8A and B). On the other hand, ligand RMSD (Lig fit Prot) remains consistently below ~ 2.0 Å with minimal fluctuation for **9** and ~ 2.4 Å for **18**. Neither ligand diffuses from the binding site, where **9** appears slightly more rigid, while **18** has a bit more breathing room, but remains fully bound. The Root Mean Square Fluctuation (RMSF) plot shows peaks indicating the enzyme’s most flexible regions (Fig. 8C and D).

*α*‑Helices (red backgrounds) and *β*‑strands (blue backgrounds) generally correspond to regions with low RMSF values, indicating structural rigidity. Peaks of high fluctuation are typically associated with loop regions, most prominently at the *N*‑ and C‑termini. Residues interacting with the ligand are marked with green bars; these residues generally exhibit reduced fluctuations, reflecting stabilization by the ligand and confirming the presence of a stable binding pocket.

In line with the docking analysis, the enzyme-ligand **9** interaction likely shows a high prevalence of hydrogen bonds and strong hydrophobic contacts, especially with TRP (Fig. 8E). While the ligand **18** relies heavily on hydrophobic packing within AChE and on water bridges, suggesting that the fit is not perfectly complementary and that water molecules act as “adapters” to facilitate the interactions (Fig. 8F). As shown in Fig. 8G and H, both ligands appear to maintain a stable radius of gyration (rGyr), indicating that they do not undergo folding or unfolding during the simulation. Compound **18** shows a slightly higher rGyr, suggesting a more extended conformation. A stable solvent-accessible surface area (SASA) indicates that both ligands remain consistently buried within the pocket.

Monitoring of the polar surface area (PSA) further confirms that the polar groups of the ligands remain engaged in hydrogen‑bond interactions rather than flipping into the solvent, as observed for compound **9**. In contrast, the reliance of compound **18** on water bridges suggests that part of its polar surface area is satisfied by water molecules rather than direct protein contacts (Fig. 8G and H). Notably, AChE inhibitors such as donepezil and galantamine use hydrogen bonds with aromatic residues in the active site while also relying on hydrophobic interactions for their effectiveness. In MD simulations, hydrophobic ligands often bind more stably, although they may not exhibit as strong an affinity as polar ligands^76^.

Similarly, MD simulations of versicolactone B (**11**) and quercetin‑3‑gentiobioside (**15**), selected based on their higher docking scores against BACE‑1 (PDB: 4LXM), revealed stable enzyme–ligand interactions. The RMSD of the enzyme fluctuated by ~ 2–3 Å and stabilized after ~ 100 ns in the presence of ligand **11**, whereas in the presence of ligand **15**, the RMSD remained stable with fluctuations of ~ 2–2.5 Å.

**Fig. 9:**
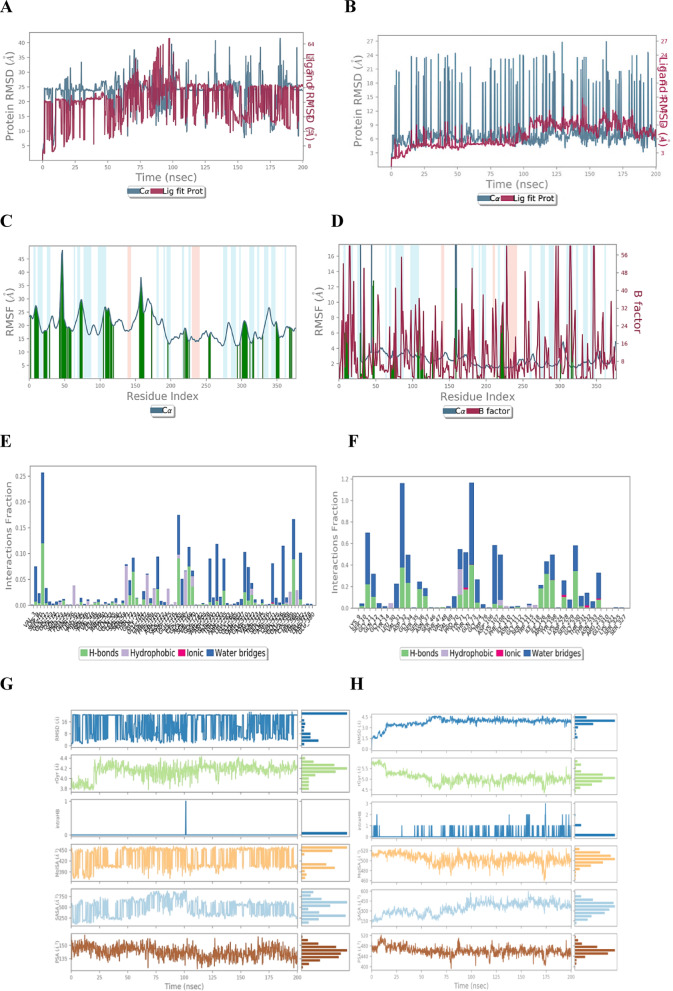
MD simulations for the interactions of EtOAc *A. oryzae* compound (**11)** (**A**, **C**, **E**, and **G**) and MeOH *E. grusonii* spines compound (**15)** (**B**, **D**, **F**, and **H**) against BACE-1 (PDB: 4LXM).

Both simulations reached equilibrium, and the protein remained structurally stable (Fig. 9A and B). Higher fluctuations (~ 4–6 Å), suggesting partial mobility, were observed for ligand **11**, whereas ligand **15** exhibited lower fluctuations (~ 2–3 Å), indicating tighter binding. Peaks corresponding to flexible loops and termini were evident in the enzyme RMSF, with ligand **11** forming contacts across residues such as LYS9, TYR14, GLN25, HIS49, and TRP115. In contrast, ligand **15** showed stronger contacts within similar flexible regions, particularly with residues including GLY11, TYR71, ASP106, and ARG128 (Fig. 9C and D).

The protein–ligand contact analysis revealed approximately eight total contacts for ligand **11**, dominated by hydrophobic interactions with occasional hydrogen bonds. In contrast, ligand **15** formed around twenty total contacts, characterized by strong hydrogen bonding and water bridges (Fig. 9E and F). Both ligands maintained a consistent rGyr and stable burial within the binding pocket. The stable PSA further suggests that the polar groups of the ligands are engaged in hydrogen bonding with the protein and do not flip into the solvent.

## Conclusion

This study explored the neuroprotective potential of *Echinocactus grusonii* (spines and stems) and its associated endophytic fungus *Aspergillus oryzae*. Extracts from both sources exhibited strong inhibitory activity against AChE and BACE‑1, two key enzymes implicated in Alzheimer’s disease. Chemical analysis revealed that these extracts are rich in phenolics, flavonoids, and alkaloids. Detailed profiling using GC and UPLC–MS/MS identified multiple bioactive metabolites, several of which demonstrated strong binding affinities to AChE and BACE‑1 in molecular docking studies. Molecular dynamics simulations further validated the stability of these interactions.

Overall, the findings suggest that *E. grusonii* and its endophytic fungus *A. oryzae* represent promising sources of multi‑target natural inhibitors for Alzheimer’s disease. The proposed dual‑target neuroprotective potential is supported by experimental enzyme inhibition assays, computational analyses, and previously reported neuroprotective activities of several identified metabolites. Nevertheless, further cellular and in vivo investigations are required to establish comprehensive biological validation and safety profiles of these bioactive compounds.

## Supplementary Information

Below is the link to the electronic supplementary material.

Supplementary Material 1

## Data Availability

The data generated and evaluated in this manuscript are included in the published article and its supplementary materials.

## References

[1] Heraiz, A. A. et al. Dual inhibition of acetylcholinesterase and *β*-secretase by metabolites from *Echinocactus grusonii* Hildm.: *in silico* and *in vitro* investigations. *RSC Adv.* **15**, 49178–49188 (2025). 41384075 10.1039/d5ra07497e PMC12690644

[2] Conte, G., Minhós-Yano, I., Moraes, E. M., Costa, D., Franco, F. F. & F. B. & Secondary metabolites of Cactaceae: current knowledge and perspectives. *Discov Plants*. **2**, 243 (2025).

[3] Hultine, K. R. et al. The role of botanical gardens in the conservation of Cactaceae. 10.1093/biosci/biw128 (2016).

[4] Huang, F. & Guo, W. Structural and mechanical properties of the spines from *Echinocactus grusonii* cactus. *J. Mater. Sci.* **48**, 5420–5428 (2013).

[5] Oonsivilai, R., Chaijareonudomroung, N., Huantanom, Y. & Oonsivilai, A. Extraction condition of *Echinocactus grusonii*. *World Acad. Sci. Eng. Technol.* **46**, 77–80 (2010).

[6] Zuo, Y., Hu, Q., Zhang, K. & He, X. Host and tissue affiliations of culturable endophytic fungi associated with xerophytic plants in the desert region of Northwest China. *Agronomy*10.3390/agronomy12030727 (2022).

[7] Hussain, F. et al. Promising thiazolidinedione-thiazole based multi-target and neuroprotective hybrids for Alzheimer’s disease: Design, synthesis, *in-vitro*, *in-vivo* and *in-silico* studies. *Eur. J. Med. Chem.* **287**, 117327 (2025). 39914143 10.1016/j.ejmech.2025.117327

[8] Kazeem, M. I., Abdulsalam, R. A., Mellem, J. J. & Sabiu, S. Lemongrass (*Cymbopogon citratus*) infusions exhibit neuroprotective properties: Evidence from *in vitro* and *in silico* studies. *Food Biosci.* **68**, 106355 (2025).

[9] Fissel, J. A. & Farah, M. H. The influence of BACE1 on macrophage recruitment and activity in the injured peripheral nerve. *J. Neuroinflamm.*10.1186/s12974-021-02121-2 (2021). 10.1186/s12974-021-02121-2 PMC7962400 33722254

[10] Colovic, M. B., Krstic, D. Z., Lazarevic-Pasti, T. D., Bondzic, A. M. & Vasic, V. M. Acetylcholinesterase inhibitors: Pharmacology and toxicology. *Curr. Neuropharmacol.*10.2174/1570159X11311030006 (2013). 24179466 10.2174/1570159X11311030006 PMC3648782

[11] Chang, C. C., Yang, M. H., Wen, H. M. & Chern, J. C. Estimation of total flavonoid content in propolis by two complementary colometric methods. *J. Food Drug Anal.*10.38212/2224-6614.2748 (2002).

[12] Sembiring, E. N., Elya, B. & Sauriasari, R. Phytochemical screening, total flavonoid and total phenolic content and antioxidant activity of different parts of *Caesalpinia bonduc* (L.) Roxb.. *Pharmacogn. J.*10.5530/pj.2018.1.22 (2018).

[13] Shamsa, F., Monsef, H., Ghamooshi, R. & Verdian-rizi, M. Spectrophotometric determination of total alkaloids in some Iranian medicinal plants. *Thai J. Pharm. Sci.* **32**, 17–20 (2008).

[14] Talaat, A. N. et al. Targeted neuroprotection in sporadic Alzheimer’s disease: UPLC-ESI-MS/MS profiling and bilosome-mediated delivery of *Crateva magna* and its endophytic fungal extracts. *Phytochem. Anal.*10.1002/pca.3540 (2025). 40420219 10.1002/pca.3540

[15] Varela, M. C., Arslan, I., Reginato, M. A., Cenzano, A. M. & Luna, M. V. Phenolic compounds as indicators of drought resistance in shrubs from Patagonian shrublands (Argentina). *Plant Physiol. Biochem.*10.1016/j.plaphy.2016.03.014 (2016). 27017434 10.1016/j.plaphy.2016.03.014

[16] Bhambhani, S., Kondhare, K. R. & Giri, A. P. Diversity in chemical structures and biological properties of plant alkaloids. *Molecules*10.3390/molecules26113374 (2021). 34204857 10.3390/molecules26113374 PMC8199754

[17] Viera-Escareño, V. et al. Alkaloid and nitrogenated compounds from different sections of *Coryphantha macromeris* plants and callus cultures. *Appl. Sci.* **13**, 9947 (2023).

[18] Cassels, B. K. Alkaloids of the cactaceae? The classics. *Nat Prod. Commun* **14**, (2019).

[19] Zou, J. et al. UPLC-Q-TOF-MS/MS analysis on the chemical composition of malts under different germination cycles and prepared with different processing methods. *Fitoterapia*10.1016/j.fitote.2022.105313 (2023). 36179899 10.1016/j.fitote.2022.105313

[20] Wang, H. et al. Rapid discovery and global characterization of chemical constituents and rats metabolites of *Phellodendri amurensis* cortex by ultra-performance liquid chromatography-electrospray ionization/quadrupole-time-of-flight mass spectrometry coupled with pattern recognition approach. *Analyst*10.1039/c3an36902a (2013). 23608925 10.1039/c3an36902a

[21] Globisch, D. et al. Onchocerca volvulus-neurotransmitter tyramine is a biomarker for river blindness. *Proc. Natl. Acad. Sci. U S A*. **110**, 4218–4223 (2013). 23440222 10.1073/pnas.1221969110 PMC3600455

[22] Cao, X., Lin, X., Wu, C., Zhang, M. & Wang, M. Green extraction-assisted pseudo-targeted profile of alkaloids in lotus seed epicarp based on UPLC-QTOF MS with IDA. *Foods*10.3390/foods11071056 (2022). 35407146 10.3390/foods11071056 PMC8997499

[23] Khalid, M., Bilal, M. & Huang, D. F. Role of flavonoids in plant interactions with the environment and against human pathogens — A review. *J. Integr. Agric.*10.1016/S2095-3119(19)62555-4 (2019).

[24] Karaźniewicz-Łada, M. et al. Application of UPLC-MS/MS method for analysis of apigenin, apigenin 7-glucoside and chlorogenic acid in goat serum. *Chromatographia*10.1007/s10337-023-04250-7 (2023).

[25] Zheng, Y. et al. Integrating pharmacology and gut microbiota analysis to explore the mechanism of Citri Reticulatae Pericarpium against reserpine-induced spleen deficiency in rats. *Front. Pharmacol.*10.3389/fphar.2020.586350 (2020). 33192528 10.3389/fphar.2020.586350 PMC7606944

[26] Elshabrawy, M. O. et al. Phytochemical profiling, cytotoxic effect, and *in vitro* anti-diabetic assessment of *Asparagus horridus* L. with *in silico* insights of Brassicin. *Egypt. J. Chem.* **68**, 583–600 (2025).

[27] Elbana, R. M., Taie, H. A. A., Moustafa, A. M. Y. & Marzouk, M. LC-MS/MS analyses of *Khaya grandifoliola* and *in vitro* antioxidant activity and cytotoxicity of *Khaya senegalensis* and *Khaya grandifoliola* against Ehrlich ascites carcinoma cells. *Egypt. J. Chem.*10.21608/ejchem.2024.259569.9124 (2024).

[28] Xu, F., Liu, Y., Zhang, Z., Yang, C. & Tian, Y. Quasi-MSn identification of flavanone 7-glycoside isomers in Da Chengqi Tang by high performance liquid chromatography-tandem mass spectrometry. *Chin. Med.*10.1186/1749-8546-4-15 (2009). 19630957 10.1186/1749-8546-4-15 PMC2722651

[29] March, R. E. & Miao, X. S. A fragmentation study of kaempferol using electrospray quadrupole time-of-flight mass spectrometry at high mass resolution. *Int. J. Mass Spectrom.*10.1016/j.ijms.2003.10.008 (2004).

[30] Fathoni, A., Candraditya, A. N. & Rudiana, T. Antioxidant activity and identification of flavonoid compounds in Patat leaves (*Phrynium capitatum*) ethyl acetate extract. *J. Pendidik. Kim.*10.24114/jpkim.v14i3.40595 (2022).

[31] He, Z. H., Liu, M., Ren, J. X. & Ouyang, D. W. Structural characterization of chemical compounds based on their fragmentation rules in *Sophorae fructus* by UPLC-QTOF-MS/MS. *Pharm. Front.* **4**, e162–e178 (2022).

[32] Kaur, J., Dhiman, V., Bhadada, S., Katare, O. P. & Ghoshal, G. LC/MS guided identification of metabolites of different extracts of *Cissus quadrangularis*. *Food Chem. Adv.* **1**, 100084 (2022).

[33] Ma, Y. L., Van Den Heuvel, H. & Claeys, M. Characterization of 3-methoxyflavones using fast-atom bombardment and collision-induced dissociation tandem mass spectrometry. *Rapid Commun. Mass Spectrom.* **13** (1999). 10.1002/(SICI)1097-0231(19991015)13:19<1932::AID-RCM735>3.0.CO;2-W 10487940

[34] El-Zahar, H. et al. UPLC-PDA-MS/MS profiling and healing activity of polyphenol-rich fraction of *Alhagi maurorum* against oral ulcer in rats. *Plants (Basel, Switzerland)*10.3390/plants11030455 (2022). 35161436 10.3390/plants11030455 PMC8838639

[35] Gholamalipour Alamdari, E. & Taleghani, A. New bioactive compounds characterized by liquid chromatography–mass spectrometry and gas chromatography–mass spectrometry in hydro-methanol and petroleum ether extracts of *Prosopis farcta* (Banks & Sol.) J. F. Macbr weed. *J. Mass Spectrom.*10.1002/jms.4884 (2022). 36128672 10.1002/jms.4884

[36] Schmidt, S. et al. Identification of complex, naturally occurring flavonoid glycosides in kale (*Brassica oleracea* var. *sabellica*) by high-performance liquid chromatography diode-array detection/electrospray ionization multi-stage mass spectrometry. *Rapid Commun. Mass Spectrom.*10.1002/rcm.4605 (2010). 20552580 10.1002/rcm.4605

[37] Vrhovsek, U., Masuero, D., Palmieri, L. & Mattivi, F. Identification and quantification of flavonol glycosides in cultivated blueberry cultivars. *J. Food Compos. Anal.*10.1016/j.jfca.2011.04.015 (2012).

[38] Truchado, P., Vit, P., Heard, T. A., Tomás-Barberán, F. A. & Ferreres, F. Determination of interglycosidic linkages in O-glycosyl flavones by high-performance liquid chromatography/photodiode-array detection coupled to electrospray ionization ion trap mass spectrometry. Its application to *Tetragonula carbonaria* honey from Australia. *Rapid Commun. Mass Spectrom.*10.1002/rcm.7184 (2015). 26407309 10.1002/rcm.7184

[39] Yin, R. et al. UFLC-MS/MS method for simultaneous determination of luteolin-7-*O*-gentiobioside, luteolin-7-*O-β*-d-glucoside and luteolin-7-*O-β*-d-glucuronide in beagle dog plasma and its application to a pharmacokinetic study after administration of traditional Chinese medicinal preparation: Kudiezi injection. *J. Pharm. Biomed. Anal.* **72**, (2013). 10.1016/j.jpba.2012.09.028 23146236

[40] Lin, L. C., Pai, Y. F. & Tsai, T. H. Isolation of luteolin and luteolin-7-O-glucoside from Dendranthema morifolium Ramat Tzvel and their pharmacokinetics in rats. in *J. Agric. Food Chem.* **63** (2015). 10.1021/jf505848z 25625345

[41] Rodríguez-Medina, I. C., Segura-Carretero, A. & Fernández-Gutiérrez, A. Use of high-performance liquid chromatography with diode array detection coupled to electrospray-Qq-time-of-flight mass spectrometry for the direct characterization of the phenolic fraction in organic commercial juices. *J. Chromatogr. A*10.1016/j.chroma.2009.04.022 (2009). 19409569 10.1016/j.chroma.2009.04.022

[42] Jaiswal, R., Müller, H., Müller, A., Karar, M. G. E. & Kuhnert, N. Identification and characterization of chlorogenic acids, chlorogenic acid glycosides and flavonoids from *Lonicera henryi* L. (Caprifoliaceae) leaves by LC-MSn. *Phytochemistry*10.1016/j.phytochem.2014.08.023 (2014). 25236695 10.1016/j.phytochem.2014.08.023

[43] Valgimigli, L., Gabbanini, S., Matera, R. & Chapter 26. Analysis of Maltose and Lactose by U-HPLC-ESI-MS/MS. 10.1039/9781849734929-00443 (2013).

[44] Jabbar, A., Hamzah, H., Windarsih, A., Pratiwi, S. U. T. & Rohman, A. LC-MS analysis, antioxidant and anti-inflamatory activity, isolation of secondary metabolite of ethanol extract stem of *Etlingera rubroloba* AD Poulsen. *Case Stud. Chem. Environ. Eng.* **10**, 100780 (2024).

[45] Hamed, A. R., El-Hawary, S. S., Ibrahim, R. M., Abdelmohsen, U. R. & El-Halawany, A. M. Identification of chemopreventive components from halophytes belonging to *Aizoaceae* and *Cactaceae* through LC/MS—bioassay guided approach. *J. Chromatogr. Sci.* **59**, 618–626 (2021). 33352581 10.1093/chromsci/bmaa112

[46] Lolok, N., Sumiwi, S. A., Sahidin, I. & Levita, J. Stigmasterol isolated from the ethyl acetate fraction of Morinda citrifolia fruit (using the bioactivity‑guided method) inhibits α‑amylase activity: In vitro and in vivo analyses. *World Acad. Sci. J.* **5**, 25 (2023).

[47] Chen, J. J. et al. Highly oxygenated constituents from a marine alga-derived fungus *Aspergillus giganteus* NTU967. *Mar. Drugs*. **18**, 303 (2020). 32517237 10.3390/md18060303 PMC7374281

[48] Rukachaisirikul, V. et al. γ-butyrolactone, cytochalasin, cyclic carbonate, eutypinic acid, and phenalenone derivatives from the soil fungus *Aspergillus* sp. PSU-RSPG185. *J. Nat. Prod.*10.1021/np500324b (2014). 25375978 10.1021/np500324b

[49] He, P. et al. Three new phenyl ether derivatives from *Aspergillus carneus* HQ889708. *Helv. Chim. Acta*. **98**, 819–822 (2015).

[50] Baccile, J. A. et al. Plant-like biosynthesis of isoquinoline alkaloids in *Aspergillus fumigatus*. *Nat. Chem. Biol.* **12**, 419–424 (2016). 27065235 10.1038/nchembio.2061 PMC5049701

[51] Wang, L. et al. Secoyanhusamine A, an oxidatively ring-opened isoquinoline inner salt from Corydalis yanhusuo. *Front. Chem.* **9**, 831173 (2022). 35178381 10.3389/fchem.2021.831173 PMC8843934

[52] Mouafo Talontsi, F., Kongue Tatong, M. D., Dittrich, B., Douanla-Meli, C. & Laatsch, H. Structures and absolute configuration of three α-pyrones from an endophytic fungus *Aspergillus niger*. *Tetrahedron*10.1016/j.tet.2013.05.098 (2013).

[53] Phainuphong, P. et al. γ-Butenolide and furanone derivatives from the soil-derived fungus *Aspergillus sclerotiorum* PSU-RSPG178. *Phytochemistry* **137**, 165–173 (2017). 28228227 10.1016/j.phytochem.2017.02.008

[54] Tang, S. et al. Discovery and characterization of a PKS-NRPS hybrid in *Aspergillus terreus* by genome mining. *J. Nat. Prod.* **83**, 473–480 (2020). 32077283 10.1021/acs.jnatprod.9b01140

[55] Wu, Z. et al. Antioxidative phenolic compounds from a marine-derived fungus *Aspergillus versicolor*. *Tetrahedron* **72**, 50–57 (2016).

[56] Qi, C. et al. Terrusnolides A-D, new butenolides with anti-inflammatory activities from an endophytic *Aspergillus* from *Tripterygium wilfordii*. *Fitoterapia* **130**, 134–139 (2018). 30165179 10.1016/j.fitote.2018.08.021

[57] Xu, Y. et al. Kojic acid derivatives and sesquiterpenes from the *Aspergillus flavus* GZWMJZ-288, a fungal endophyte of* garcinia multiflora*. *Nat. Prod. Commun.* **13** (2018).

[58] Zhou, M. et al. Antiviral butyrolactones from the endophytic fungus *Aspergillus versicolor*. *Planta Med.* **81**, 235–240 (2015). 25590371 10.1055/s-0034-1396153

[59] Zhang, X. Q. et al. Design, semisynthesis, α-glucosidase inhibitory, cytotoxic, and antibacterial activities of *p*-terphenyl derivatives. *Eur. J. Med. Chem.* **146**, 232–244 (2018). 29407953 10.1016/j.ejmech.2018.01.057

[60] Jouini, M. et al. Phytochemical analysis, neuroprotective, anticholinesterase, cytotoxic and catalase potentials of *Opuntia microdasys* var. rufida and *Opuntia leptocaulis*. *Chem. Afr.* **4**, 285–298 (2021).

[61] Agrawal, M. et al. Neuroprotective action of hordenine against the aluminium chloride (AlCl3) induced Alzheimer’s diseases & associated memory impairment in experimental rats. *Pharmacol. Res. - Mod. Chin. Med.* **12**, 100492 (2024).

[62] Liu, R. et al. The flavonoid apigenin protects brain neurovascular coupling against amyloid-*β*25-35-induced toxicity in mice. *J. Alzheimers Dis.* **24**, 85–100 (2011). 21297270 10.3233/JAD-2010-101593

[63] Zhang, N., Nao, J. & Dong, X. Efficacy and safety of natural apigenin treatment for Alzheimer’s disease: Focus on *in vivo* research advancements. *Curr. Neuropharmacol.* **23**, 728–754 (2024). 10.2174/1570159X23666241211095018 PMC12163474 39665306

[64] Mohammadkhanizadeh, A. et al. Protective effects of apigenin in neurodegeneration: An update on the potential mechanisms. *Brain Disorders*. 10.1016/j.dscb.2025.100189 (2025).

[65] Elreedy, H. A., Elfiky, A. M., Mahmoud, A. A., Ibrahim, K. S. & Ghazy, M. A. Neuroprotective effect of quercetin through targeting key genes involved in aluminum chloride induced Alzheimer’s disease in rats. *Egypt. J. Basic Appl. Sci.*10.1080/2314808X.2022.2164136 (2023).

[66] Olennikov, D. N. et al. Isorhamnetin and quercetin derivatives as anti-acetylcholinesterase principles of marigold (*Calendula officinalis*) flowers and preparations. *Int. J. Mol. Sci.* **18**, 1685 (2017). 28767066 10.3390/ijms18081685 PMC5578075

[67] Atoki, A. V., Aja, P. M., Shinkafi, T. S., Ondari, E. N. & Awuchi, C. G. Naringenin: its chemistry and roles in neuroprotection. *Nutr. Neurosci.*10.1080/1028415X.2023.2243089 (2024). 37585716 10.1080/1028415X.2023.2243089

[68] Jin, S., Zhang, L. & Wang, L. Kaempferol, a potential neuroprotective agent in neurodegenerative diseases: From chemistry to medicine. *Biomed. Pharmacotherapy*. 10.1016/j.biopha.2023.115215 (2023). 10.1016/j.biopha.2023.115215 37494786

[69] Jamali-Raeufy, N., Baluchnejadmojarad, T., Roghani, M., keimasi, S. & goudarzi, M. Isorhamnetin exerts neuroprotective effects in STZ-induced diabetic rats via attenuation of oxidative stress, inflammation and apoptosis. *J. Chem. Neuroanat.*10.1016/j.jchemneu.2019.101709 (2019). 31698018 10.1016/j.jchemneu.2019.101709

[70] Kwon, S. H. et al. Neuroprotective effects of chlorogenic acid on scopolamine-induced amnesia via anti-acetylcholinesterase and anti-oxidative activities in mice. *Eur. J. Pharmacol.* **649**, 210–217 (2010). 20854806 10.1016/j.ejphar.2010.09.001

[71] Nguyen, V. et al. Chlorogenic acid: a systematic review on the biological functions, mechanistic actions, and therapeutic potentials. 10.3390/nu16070924 (2024). 10.3390/nu16070924 PMC11013850 38612964

[72] Ferreira De Freitas, R. & Schapira, M. A systematic analysis of atomic protein-ligand interactions in the PDB. *Medchemcomm*10.1039/C7MD00381A (2017). 29308120 10.1039/c7md00381a PMC5708362

[73] Chen, K. & Kurgan, L. Investigation of atomic level patterns in protein-small ligand interactions. *PLoS One*10.1371/journal.pone.0004473 (2009). 19221587 10.1371/journal.pone.0004473 PMC2637420

[74] Zacharias, N. & Dougherty, D. A. Cation-π interactions in ligand recognition and catalysis. *Trends Pharmacol. Sci.*10.1016/S0165-6147(02)02027-8 (2002). 12084634 10.1016/s0165-6147(02)02027-8

[75] Wilson, K. A., Kellie, J. L. & Wetmore, S. D. DNA-protein π-interactions in nature: Abundance, structure, composition and strength of contacts between aromatic amino acids and DNA nucleobases or deoxyribose sugar. *Nucleic Acids Res.*10.1093/nar/gku269 (2014). 24744240 10.1093/nar/gku269 PMC4041443

[76] Reynoso-García, M. F., Nicolás-Álvarez, D. E., Tenorio-Barajas, A. Y. & Reyes-Chaparro, A. Structural bioinformatics applied to acetylcholinesterase enzyme inhibition. *Int. J. Mol. Sci.*10.3390/ijms26083781 (2025). 40332446 10.3390/ijms26083781 PMC12028328

