# Supplementary material for: Therapeutic potential of Echinocactus grusonii and its associated endophytic fungus Aspergillus oryzae in neuroprotection: in vitro and in silico investigations
Source: Sci Rep. 2026 Jul 29;16:23556. doi: 10.1038/s41598-026-62565-5 (PMC13421660; doi:10.1038/s41598-026-62565-5)
Supplement: Supplementary file 1 — Supplementary Material 1 [file 41598_2026_62565_MOESM1_ESM.docx]

**Supplementary Information**

**Therapeutic potential of *Echinocactus grusonii* and its associated endophytic fungus *Aspergillus oryzae* in neuroprotection: *In vitro* and *in silico* investigations**

Ahmed A. Heraiz ^1*^[^,0000-0002-4829-9860^](https://orcid.org/0000-0002-4829-9860) , Ahmed Othman ^1*,^ [^0000-0002-2827-7621^](https://orcid.org/0000-0002-2827-7621), Amr Farouk ^2^, Mostafa M. Hegazy^1,^ [^0000-0001-8975-9424^,](https://orcid.org/0000-0001-8975-9424) Abd El-Salam I. Mohammed^1,^ [^0000-0002-5554-9602^](https://orcid.org/0000-0002-5554-9602) and Atef A. El-Hela^1^, [^0009-0006-6746-4282^](https://orcid.org/0009-0006-6746-4282)

^1^Department of Pharmacognosy and Medicinal Plants, Faculty of Pharmacy, Al-Azhar University, Cairo, 11884, Egypt

^2^Flavour and Aroma Chemistry Department, National Research Centre, Dokki, Giza 12622, Egypt

*** Correspondence:**

Ahmed A. Heraiz; ahmedheraiz.2@azhar.edu.eg; <https://orcid.org/0000-0002-4829-9860>

Ahmed Othman; [ah.othman@azhar.edu.eg](mailto:ah.othman@azhar.edu.eg); <https://orcid.org/0000-0002-2827-7621>

**Abstract**

The Golden Barrel Cactus (*Echinocactus grusonii*, syn. *Kroenleinia grusonii*), native to Mexico, is well known in horticulture but remains insufficiently studied with respect to its chemical composition. This study examined the chemical profile and neuroprotective potential of *Echinocactus grusonii* spines and its endophytic fungus, *Aspergillus oryzae*. The spines contained markedly higher levels of phenolics (293 mg gallic acid/g DW extract), flavonoids (132 mg quercetin/g DW extract), and alkaloids (14.5 mg atropine/g DW extract) compared to the stem. Untargeted metabolomics (UPLC-HRMS/MS) tentatively identified 27 metabolites in the spines and 14 in the fungal extract. Both extracts exhibited potent, dose-dependent inhibition of acetylcholinesterase (AChE) and β-secretase (BACE-1). The spines extract showed IC₅₀ values of 0.362 µg/mL (AChE) and 0.425 µg/mL (BACE-1), while the fungal extract was more effective against AChE (IC₅₀ = 0.255 µg/mL). *In silico* docking was performed for all identified compounds, and molecular dynamics simulation of the top-scoring candidates demonstrated stable interactions with the target enzymes. These findings highlight the spines of *Echinocactus grusonii* and their associated endophytes as promising dual-target neuroprotective sources for the management of neurodegenerative disorders.

**Key words:** Cactaceae, Kroenleinia grusonii, Neuroprotection, Computational Studies, Liquid chromatography–mass spectrometry (LC–MS), Bioactive metabolite

- 1. **Molecular Docking Study**

ChemDraw software was used to create a 2D diagram in sdf format of the ligand structures identified by UPLC-MS/MS analysis of the methanol and EtOAc extracts from *Echinocactus grusonii* spines and *Aspergillus oryzae*. After converting the 2D structure to 3D in Avogadro 1.2.0, optimization was performed using the MMFF94 force field. The structures of donepezil and curcumin were retrieved from PubChem (http://pubchem.ncbi.nlm.nih.gov/, accessed January 7, 2026) as positive standards. Crystal structures of AChE (PDB ID: 4M0E) and BACE1 (PDB ID: 4LXM) were obtained from the Protein Data Bank (PDB) (https://www.rcsb.org, accessed on January 6, 2026). For the validation of the molecular docking protocol, Discovery Studio Visualizer and CB-Dock2 were used to identify the binding sites for 4M0E (Centre: X = -14, Y = -42, Z = 27, and Size: X = 18, Y = 14, Z = 18) and for 4LXM (Centre: X = 30, Y = 4, Z = 21, and Size: X = 16, Y = 17, Z = 30). The docking method was validated by redocking co-crystallized ligands (1YL and 1YU) using AutoDock 1.5.6 and Vina, with RMSD values < 2.0 Å (0.5637 and 1.194 Å, respectively), as shown in Figure S1. CB-Dock2 was used for docking (http://clab.labshare.cn/cb-dock/php/, accessed on January 6-7, 2026), according to ^1^. The Discovery Studio program ver 24.1.0.23298 visualized profiles for the docked complexes ^2^.

4M0E-1YL: 0.5637A


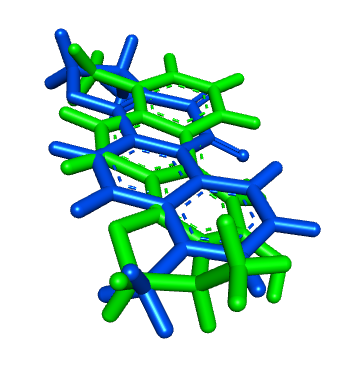


4LXM-1YU: 1.194A


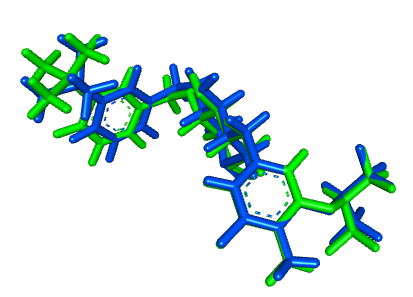


**Figure S1.** The validation of docking performance by AutoDock Vina for enzymes and co-crystal ligands (The cocrystallized and docked ligands are depicted as sticks in green and blue, respectively).

- 1. **Molecular Dynamics Simulation (MD)**

MD simulations were performed for the ligands 9 and 11 of *Aspergillus oryzae* and 15 and 18 of *Echinocactus grusonii* spines with 4M0E and 4LXM using the Desmond package (Schrödinger LLC, NY, USA). Following equilibration with a 1‑ps as a relaxation step, simulations were performed for 200 ns in the NPT ensemble at 310 K and 1 bar. The OPLS_2005 force field was applied, and long‑range electrostatics were treated using the particle‑mesh Ewald method with a 9.0 Å cutoff. Water molecules were modelled with the SPC representation. Pressure and temperature were controlled using the Martyna–Tuckerman–Klein barostat and Nosé–Hoover thermostat, respectively. Non‑bonded interactions were integrated via r‑RESPA, and trajectories were saved every 4.8 ps. Ligand preparation was carried out in AMBER 14, employing the ff99 force field for energy minimization, counterion addition, and MD simulations in explicit TIP4P water ^3^. Geometries were further refined using density functional theory (DFT) with a 6‑31G basis set. To ensure adequate conformational sampling, independent simulations were conducted with a 0.001 ps time step. System stability was confirmed, and trajectory analyses were performed using cpptraj from AMBER Tools ^4^.

**Table S1:** GC-MS analysis of Echinocactus grusonii spines extract.

| **Compound** | **R_t_** | **Molecular weight** | **Molecular Formula** | **Percent (%)** |
| --- | --- | --- | --- | --- |
| 3-oxo-20-methy l-11-*α*-hydroxy conanine-1,4-diene | 6.09 | 341 | C_22_H_31_NO_2_ | 2.43 |
| Octadecenyloxyethanol | 6.46 | 312 | C_20_H_40_O_2_ | 3.66 |
| *O*-benzyl serine | 6.70 | 195 | C_10_H_13_NO_3_ | 0.47 |
| 3*α*-hydroxy-6-benzyl-6 azacholest-4-en-7-one | 7.31 | 491 | C_33_H_49_NO_2_ | 1.85 |
| 9-octadecenoic acid, (2-phenyl-1,3-dioxolan-4-yl) methyl ester | 8.7 | 444 | C_28_H_44_O_4_ | 8.53 |
| Propanoic acid, 2-(3-acetoxy-4,4,14-trimethylandrost-8-en-17-yl)- | 9.63 | 430 | C_27_H_42_O_4_ | 0.66 |
| Glycerol 1-palmitate | 9. 87 | 330 | C_19_H_38_O_4_ | 19.96 |
| Secoisolariciresinol | 11.72 | 311 | C_20_H_23_NO_4_ | 4.38 |
| 2,3-dihydroxypropyl stearate | 12.88 | 358 | C_21_H_42_O_4_ | 15.33 |
| Astaxanthin | 13.45 | 596 | C_40_H_52_O_4_ | 1.86 |
| 2-acetyl-3-(2-cinnamido) ethyl-7-methoxyindole | 6.09 | 362 | C_22_H_22_N_2_O_3_ | 2.43 |

**Table S2**: Identified compounds in the spines’ methanol extract via UPLC-ESI-MS/MS analysis

| **No.** | ***t_R_***  **(min)** | **Adduct**  **ion** | **Observed (Calculated)**  ***m/z*** | **Mass error**  **(ppm)** | **MS/MS**  ***m/z*** | **Molecular**  **formula** | **Proposed compound** | Ref. |
| --- | --- | --- | --- | --- | --- | --- | --- | --- |
|  | 0.62 | [M+H] ^+^ | 166.1233  (166.1232) | 0.6 | 121.0675 (M+H-N(CH_3_)_2_)  103.0557 (M+H-N(CH_3_)_2_-OH) | C_10_H_15_NO | hordenine | ^5^ |
|  | 2.35 | [M] ^+^ | 180.1383  (180.1394) | 6.10 | 121[M-C_3_H_9_N] ^+^ | C_11_H_18_NO | Candicine | ^6^ |
|  | 0.60 | [M+H] ^+^ | 180.1026  (180.1025) | 0.56 | 166[M+H-CH_2_] ^+^  121[M+H-C_2_H_5_NO] ^+^ | C_10_H_13_NO_2_ | *N*-Acetyl tyramine | ^7^ |
|  | 0.60 | [M+H] ^+^ | 194.1172 (194.1181) | -4.64 | 180[M+H-CH_2_] ^+^  165[M+H-C_2_H_5_] ^+^ | C_11_H_15_NO_2_ | *N*-methyl-*N*-(4-hydroxyphenethyl) acetamide | ^7^ |
|  | 6.14 | [M+H] ^+^ | 314.1371  (314.1392) | -6.68 | 177 [M+H-Feruloyl] ^+^  121[M+H-Feruloyl amide] ^+^ | C_18_H_19_NO_4_ | (*E*)-*N*-feruloyl tyramine | ^8^ |
|  | 9.94 | [M+H] ^+^ | 271.0600  (271.0607) | -2.39 | 271 [M+H] ^+^  153 [A^1,3^] + | C_15_H_10_O_5_ | Apigenin | ^9^ |
|  | 7.03 | [M+H] ^+^ | 273.0758  (273.0763) | 1.83 | 273 [M+H] ^+^  153 [^1,3^A]^+^ | C_15_H_12_O_5_ | Naringenin | ^10^ |
|  | 7.16 | [M+H] ^+^ | 287.0551  (287.0555) | -1.39 | 287 [M+H] ^+^  165 [^0,2^A] ^+^  153 [^1,3^A]^+^ | C_15_H_10_O_6_ | Kaempferol | ^11^ |
|  | 4.84 | [M+H] ^+^ | 303.0502  (303.0504) | 0.66 | 303 [M+H] ^+^  201 [C_12_H_9_O_3_] ^+^  121 [C_7_H_5_O_3_] ^+^ | C_15_H_10_O_7_ | Quercetin | ^12^ |
|  | 10.08 | [M-H] ^−^ | 299.0567 | 1.9 | 284.03381 [M-H- CH_3_]^-^  151.00454 [^1,3^A] ^−^ | C_16_H_12_O_6_ | Isokaempferide | ^13^ |
|  | 7.48 | [M+H] ^+^ | 301.0711  (301.0712) | -0.99 | 301 [M+H] ^+^  286 [M+H-CH_3_] ^+^,  258 [M+H-CH_3_-CO] ^+^ |  |  | ^14^ |
|  | 7.60 | [M+H] ^+^ | 331.0816  (331.0817) | -0.42 | 331 [M+H] ^+^  316 [M+H-CH_3_] ^+^  166 [B^1,3^-CH_3_] ^+^ | C_17_H_14_O_7_ | Quercetin 3,4'(or 3')-dimethyl ether | ^15^ |
|  | 4.93 | [M+H] ^+^ | 317.0658  (17.0661) | -0.94 | 317 [M+H] ^+^  302 [M+H-CH_3_] ^+^  164 [B^1,3^] ^+^ | C_16_H_12_O_7_ | Quercetin 3'-methyl ether (Isorhamnetin) | ^16,17^ |
|  | 2.90 | [M-H] ^−^ | 609.1467 | 1.0 | 447.09501[M–H–162]^-^  285.04198[M–H–324] ^-^ | C_27_H_30_O_16_ | Kaempferol-3-gentiobioside | ^18^ |
|  | 5.42 | [M+H] ^+^  [M+Na] ^+^ | 595.1663  (595.1663)  617.1485  (617.1482) | 0.0 | 595 [M+H] ^+^  617 [M+Na] ^+^  287 [M+H-308] ^+^ | C_27_H_30_O15 | Kaempferol-3-O-rutinoside | ^18^ |
|  | 1.82 | [M-H] ^−^ | 625.1418 | 1.3 | 463.09091 [M–H–162]^-^  301.03731 [M–H–324] | C_27_H_30_O_17_ | Quercetin-3-gentiobioside | ^18,19^ |
|  | 5.77 | [M-H] ^–^ | 477.1049 | 2.2 | 315.05229 [M-H-162]^-^  300.02945 [Isorhamnetin- H- CH_3_]^-^ | C_22_H_22_O_12_ | Isorhamnetin-3-*O* glucoside | ^20^ |
|  | 5.8 | [M+H]^+^ | 479.1194  (479.1189) | 1.04 | 479 [M+H] ^+^  317 [M+H-Hexoside] ^+^  302 [M+H-Hexose-CH_3_] ^+^ | C_22_H_22_O_12_ | Isorhamnetin 3-galactoside | ^21–23^ |
|  | 5.67 | [M+H] ^+^ | 463.1242  (463.1240) | 0.43 | 463 [M+H] ^+^  301 [M-Rha-CH_3_] ^+^ | C_22_H_22_O_11_ | Isorhamnetin 3-*O*-rhamnoside | ^23^ |
|  | 5.48 | [M+H] ^+^ | 641.1724  (641.1718) | 0.93 | 641 [M+H] ^+^  317 [M+H-324] ^+^ | C_28_H_32_O_17_ | Isorhamnetin-3-*O*-(2-hexosyl) hexoside | ^24^ |
|  | 5.34 | [M-H] ^–^ | 433.1150 | 2.2 | 271.06241[M-H-162]^-^  151.00466 [^1,3^A] ^−^ | C_21_H_22_O_10_ | Naringenin 7-*O* glucoside | ^25,26^ |
|  | 2.88 | [M+H] ^+^ | 611.1613  (611.1612) | 0.16 | 611 [M+H] ^+^  287 [M+H-324] ^+^  144 | C_27_H_30_O_16_ | Luteolin-7-O-gentiobioside | ^27,28^ |
|  | 1.99 | [M-H] ^−^ | 353.0888 | 1.5 | 191.05678 (quinic acid-H) | C_16_H_18_O_9_ | Chlorogenic acid | ^29,30^ |
|  | 1.52 | [M-H] ^−^ | 355.1045 | 3.1 | 193.05169 (Ferulic acid–H) | C_16_H_20_O_9_ | Ferulic acid *β*-glucoside | ^29^ |
|  | 1.33 | [M-H] ^−^ | 153.0195 | 4.57 | 153 [M-H]^-^  135 [M-H-H_2_O]^-^  109.0292 [M-H-CO_2_] ^-^ | C_7_H_6_O_4_ | Protocatechuic acid | ^31^ |
|  | 0.56 | [M+Na] ^+^ | 365.1058  (365.1059) | -0.27 | 365 [M+Na] ^+^  203[M+Na-Galactose] ^+^ | C_12_H_22_O_11_ | Sucrose | ^32^ |
|  | 4.13 | [M+H] ^+^ | 179.0700  (179.0708) | -4.46 | 179 [M+H] ^+^  162 [M+H-H_2_O] ^+^ | C_10_H_10_O_3_ | Ferulaldehyde | ^33^ |
|  | 24.27 | [M-H] ^-^ | 413.2653 | 1.5 | 395.3687 [M-H-H_2_O] ^-^  391.2826, 359.2407  301.0747, 279.0939 | C_29_H_48_O | Stigmasterol | ^34,35^ |

**Table S3**: Identified compounds in Aspergillus oryzae EtOAc extract

| **No.** | ***t_R_***  **(min)** | **Adduct**  **ion** | **Observed (Calculated)**  ***m/z*** | **Mass error**  **(ppm)** | **Molecular**  **formula** | **Proposed compound** | **Chemical class** | **Ref.** |
| --- | --- | --- | --- | --- | --- | --- | --- | --- |
|  | 2.68 | [M+H] ^+^ | 169.0496  (169.0501) | -2.96 | C_8_H_9_O_4_ | Aspergilsmin C | Polyketide lactone | ^36^ |
|  | 6.59 | [M+H] ^+^ | 237.0720  (237.0758) | -16.02 | C_12_H_12_O_5_ | Aspergillusol B | Cyclic carbonate | ^37^ |
|  | 5.62 | [M+H] ^+^ | 238.0849  (238.0863) | -5.88 | C_15_H_11_NO_2_ | Viridicatin | Phenyl quinoline | ^38^ |
|  | 1.69 | [M+H] ^+^ | 240.1242  (240.1231) | 4.58 | C_12_H_17_NO_4_ | Campyrone C | *α*-Pyrone derivative | ^39^ |
|  | 7.07 | [M+H] ^+^ | 265.1038  (265.1071) | -12.45 | C_14_H_16_O_5_ | Aspersclerotiorone A | γ-Butenolide-furanone dimer | ^40^ |
|  | 3.18 | [M+H] ^+^ | 274.1058  (274.1074) | -5.84 | C_15_H_15_NO4 | Pyranterrone A | Isoindoles | ^41^ |
|  | 15.49 | [M+H] ^+^ | 291.0665  (291.0612) | 18.20 | C_13_H_10_N_2_O_6_ | Fumisoquin C | Isoquinoline alkaloid | ^42^ |
|  | 1.59 | [M+H] ^+^ | 293.0636  (293.0656) | -6.82 | C_14_H_12_O_7_ | Aspergilol F | Phenolic | ^43^ |
|  | 18.42 | [M+H] ^+^ | 311.1620  (311.1642) | -7.07 | C_20_H_22_O_3_ | Terrusnolide A | Butenolide | ^44^ |
|  | 2.28 | [M+H] ^+^ | 353.0827  (353.0867) | -11.32 | C_16_H_16_O_9_ | Dikojiacid A | Pyranone | ^45^ |
|  | 10.08 | [M+H] ^+^ | 409.1626  (409.1646) | 4.89 | C_24_H_24_O_6_ | Versicolactone B | Butyrolactone | ^46^ |
|  | 11.58 | [M+H] ^+^ | 437.1952  (437.1959) | -1.6 | C_26_H_28_O_6_ | Prenylterphenyllin F | Prenylated *p*-Terphenyl | ^47^ |
|  | 1.19 | [M+H] ^+^ | 441.1975  (441.202) | -10.19 | C_24_H_28_N_2_O_6_ | Aspergilline C | Highly oxygenated cyclopiazonic acid (CPA)-derived alkaloids | ^48^ |
|  | 2.27 | [M+H] ^+^ | 456.1688  (456.1667) | 4.6 | C_25_H_21_N_5_O_4_ | Versiquinazoline F | Fumi quinazoline-type alkaloid | ^49^ |

**References**

1. Liu, Y. *et al.* CB-Dock2: improved protein-ligand blind docking by integrating cavity detection, docking and homologous template fitting. *Nucleic Acids Res.* **50**, (2022).

2. Jilani, S. *et al.* Synergistic antibacterial effects of clove essential oil and eugenol with ciprofloxacin against MDR Gram-negative bacteria: *in vitro* and *in silico* approaches. *South African J. Bot.* **190**, 567–580 (2026).

3. Case, D. A. *et al.* Amber 10. (2008).

4. Kumar, B. K. *et al.* Pharmacophore based virtual screening, molecular docking, molecular dynamics and MM-GBSA approach for identification of prospective SARS-CoV-2 inhibitor from natural product databases. *J. Biomol. Struct. Dyn.* **40**, (2022).

5. Zou, J. *et al.* UPLC-Q-TOF-MS/MS analysis on the chemical composition of malts under different germination cycles and prepared with different processing methods. *Fitoterapia* **165**, (2023).

6. Wang, H. *et al.* Rapid discovery and global characterization of chemical constituents and rats metabolites of *Phellodendri amurensis* cortex by ultra-performance liquid chromatography-electrospray ionization/quadrupole-time-of-flight mass spectrometry coupled with pattern recognition approach. *Analyst* **138**, (2013).

7. Globisch, D. *et al.* Onchocerca volvulus-neurotransmitter tyramine is a biomarker for river blindness. *Proc. Natl. Acad. Sci. U. S. A.* **110**, (2013).

8. Cao, X., Lin, X., Wu, C., Zhang, M. & Wang, M. Green Extraction-Assisted Pseudo-Targeted Profile of Alkaloids in Lotus Seed Epicarp Based on UPLC-QTOF MS with IDA. *Foods* **11**, (2022).

9. Karaźniewicz-Łada, M. *et al.* Application of UPLC-MS/MS Method for Analysis of Apigenin, Apigenin 7-Glucoside and Chlorogenic Acid in Goat Serum. *Chromatographia* **86**, (2023).

10. Zheng, Y. *et al.* Integrating Pharmacology and Gut Microbiota Analysis to Explore the Mechanism of Citri Reticulatae Pericarpium Against Reserpine-Induced Spleen Deficiency in Rats. *Front. Pharmacol.* **11**, (2020).

11. March, R. E. & Miao, X. S. A fragmentation study of kaempferol using electrospray quadrupole time-of-flight mass spectrometry at high mass resolution. *Int. J. Mass Spectrom.* **231**, (2004).

12. Kaur, J., Dhiman, V., Bhadada, S., Katare, O. P. & Ghoshal, G. LC/MS guided identification of metabolites of different extracts of *Cissus quadrangularis*. *Food Chem. Adv.* **1**, (2022).

13. Xu, X. *et al.* The chemical composition of brazilian green propolis and its protective effects on mouse aortic endothelial cells against inflammatory injury. *Molecules* **25**, (2020).

14. Fathoni, A., Candraditya, A. N. & Rudiana, T. Antioxidant activity and identification of flavonoid compounds in Patat leaves (*Phrynium capitatum*) ethyl acetate extract. *J. Pendidik. Kim.* **14**, (2022).

15. Ma, Y. L., Van Den Heuvel, H. & Claeys, M. Characterization of 3-methoxyflavones using fast-atom bombardment and collision-induced dissociation tandem mass spectrometry. *Rapid Commun. Mass Spectrom.* **13**, (1999).

16. El-Zahar, H. *et al.* UPLC-PDA-MS/MS Profiling and Healing Activity of Polyphenol-Rich Fraction of *Alhagi maurorum* against Oral Ulcer in Rats. *Plants* **11**, (2022).

17. Gholamalipour Alamdari, E. & Taleghani, A. New bioactive compounds characterized by liquid chromatography–mass spectrometry and gas chromatography–mass spectrometry in hydro-methanol and petroleum ether extracts of *Prosopis farcta* (Banks & Sol.) J. F. Macbr weed. *J. Mass Spectrom.* **57**, (2022).

18. He, Z. H., Liu, M., Ren, J. X. & Ouyang, D. W. Structural Characterization of Chemical Compounds Based on Their Fragmentation Rules in *Sophorae Fructus* by UPLC-QTOF-MS/MS. *Pharm. Front.* **4**, (2022).

19. Schmidt, S. *et al.* Identification of complex, naturally occurring flavonoid glycosides in kale (*Brassica oleracea* var. *sabellica*) by high-performance liquid chromatography diode-array detection/electrospray ionization multi-stage mass spectrometry. *Rapid Commun. Mass Spectrom.* **24**, (2010).

20. Abd-El-Aziz, N. M., Hifnawy, M. S., Lotfy, R. A. & Younis, I. Y. LC/MS/MS and GC/MS/MS metabolic profiling of *Leontodon hispidulus*, *in vitro* and *in silico* anticancer activity evaluation targeting hexokinase 2 enzyme. *Sci. Rep.* **14**, (2024).

21. Shen, J. *et al.* Development of a HPLC-MS/MS Method to Determine the 13 Elements of Semen Cuscutae and Application to a Pharmacokinetic Study in Rats. *Evidence-based Complement. Altern. Med.* **2019**, (2019).

22. Schieber, A., Keller, P., Streker, P., Klaiber, I. & Carle, R. Detection of isorhamnetin glycosides in extracts of apples (*Malus domestica* cv. ‘Brettacher’) by HPLC-PDA and HPLC-APCI-MS/MS. *Phytochem. Anal.* **13**, (2002).

23. Vrhovsek, U., Masuero, D., Palmieri, L. & Mattivi, F. Identification and quantification of flavonol glycosides in cultivated blueberry cultivars. *J. Food Compos. Anal.* **25**, (2012).

24. Truchado, P., Vit, P., Heard, T. A., Tomás-Barberán, F. A. & Ferreres, F. Determination of interglycosidic linkages in O-glycosyl flavones by high-performance liquid chromatography/photodiode-array detection coupled to electrospray ionization ion trap mass spectrometry. Its application to Tetragonula carbonaria honey from Australia. *Rapid Commun. Mass Spectrom.* **29**, (2015).

25. Elbana, R. M., Taie, H. A. A., Moustafa, A. M. Y. & Marzouk, M. LC-MS/MS analyses of Khaya grandifoliola and in vitro Antioxidant Activity and Cytotoxicity of *Khaya senegalensis* and *Khaya grandifoliola* against Ehrlich Ascites Carcinoma Cells. *Egypt. J. Chem.* **67**, 509–531 (2024).

26. Xu, F., Liu, Y., Zhang, Z., Yang, C. & Tian, Y. Quasi-MSn identification of flavanone 7-glycoside isomers in Da Chengqi Tang by high performance liquid chromatography-tandem mass spectrometry. *Chin. Med.* **4**, (2009).

27. Yin, R. *et al.* UFLC-MS/MS method for simultaneous determination of luteolin-7-O-gentiobioside, luteolin-7-O-*β*-d-glucoside and luteolin-7-O-*β*-d-glucuronide in beagle dog plasma and its application to a pharmacokinetic study after administration of traditional Chinese medicinal preparation: Kudiezi injection. *J. Pharm. Biomed. Anal.* **72**, (2013).

28. Lin, L. C., Pai, Y. F. & Tsai, T. H. Isolation of Luteolin and Luteolin-7-O-glucoside from Dendranthema morifolium Ramat Tzvel and Their Pharmacokinetics in Rats. in *Journal of Agricultural and Food Chemistry* vol. 63 (2015).

29. Rodríguez-Medina, I. C., Segura-Carretero, A. & Fernández-Gutiérrez, A. Use of high-performance liquid chromatography with diode array detection coupled to electrospray-Qq-time-of-flight mass spectrometry for the direct characterization of the phenolic fraction in organic commercial juices. *J. Chromatogr. A* **1216**, (2009).

30. Jaiswal, R., Müller, H., Müller, A., Karar, M. G. E. & Kuhnert, N. Identification and characterization of chlorogenic acids, chlorogenic acid glycosides and flavonoids from *Lonicera henryi* L. (Caprifoliaceae) leaves by LC-MSn. *Phytochemistry* **108**, (2014).

31. Chen, Y. *et al.* The Analysis of *Leontopodium leontopodioides* (Willd.) Beauv. Chemical Composition by GC/MS and UPLC‐Q‐Orbitrap MS. *Int. J. Anal. Chem.* **2024**, 3525212 (2024).

32. Valgimigli, L., Gabbanini, S. & Matera, R. CHAPTER 26. Analysis of Maltose and Lactose by U-HPLC-ESI-MS/MS. in (2013). doi:10.1039/9781849734929-00443.

33. Jabbar, A., Hamzah, H., Windarsih, A., Pratiwi, S. U. T. & Rohman, A. LC-MS analysis, antioxidant and anti-inflamatory activity, isolation of secondary metabolite of ethanol extract stem of *Etlingera rubroloba* AD Poulsen. *Case Stud. Chem. Environ. Eng.* **10**, 100780 (2024).

34. Hamed, A. R., El-Hawary, S. S., Ibrahim, R. M., Abdelmohsen, U. R. & El-Halawany, A. M. Identification of chemopreventive components from halophytes belonging to Aizoaceae and Cactaceae through LC/MS—Bioassay guided approach. *J. Chromatogr. Sci.* **59**, 618–626 (2021).

35. Lolok, N., Sumiwi, S. A., Sahidin, I. & Levita, J. Stigmasterol isolated from the ethyl acetate fraction of *Morinda citrifolia* fruit (using the bioactivity‑guided method) inhibits α‑amylase activity: In vitro and in vivo analyses. *World Acad. Sci. J.* **5**, 25 (2023).

36. Chen, J. J. *et al.* Highly Oxygenated Constituents from a Marine Alga-Derived Fungus *Aspergillus giganteus* NTU967. *Mar. Drugs* **18**, (2020).

37. Rukachaisirikul, V. *et al.* γ-butyrolactone, cytochalasin, cyclic carbonate, eutypinic acid, and phenalenone derivatives from the soil fungus *Aspergillus* sp. PSU-RSPG185. *J. Nat. Prod.* **77**, (2014).

38. He, P. *et al.* Three new phenyl ether derivatives from *Aspergillus carneus* HQ889708. *Helv. Chim. Acta* **98**, (2015).

39. Mouafo Talontsi, F., Kongue Tatong, M. D., Dittrich, B., Douanla-Meli, C. & Laatsch, H. Structures and absolute configuration of three *α*-pyrones from an endophytic fungus *Aspergillus niger*. *Tetrahedron* **69**, (2013).

40. Phainuphong, P. *et al.* γ-Butenolide and furanone derivatives from the soil-derived fungus *Aspergillus sclerotiorum* PSU-RSPG178. *Phytochemistry* **137**, (2017).

41. Tang, S. *et al.* Discovery and Characterization of a PKS-NRPS Hybrid in *Aspergillus terreus* by Genome Mining. *J. Nat. Prod.* **83**, (2020).

42. Baccile, J. A. *et al.* Plant-like biosynthesis of isoquinoline alkaloids in *Aspergillus fumigatus*. *Nat. Chem. Biol.* **12**, (2016).

43. Wu, Z. *et al.* Antioxidative phenolic compounds from a marine-derived fungus *Aspergillus versicolor*. *Tetrahedron* **72**, (2016).

44. Qi, C. *et al.* Terrusnolides A-D, new butenolides with anti-inflammatory activities from an endophytic *Aspergillus* from *Tripterygium wilfordii*. *Fitoterapia* **130**, (2018).

45. Xu, Y. *et al.* Kojic acid derivatives and sesquiterpenes from the aspergillus flavus GZWMJZ-288, a fungal endophyte of *Garcinia multiflora*. *Nat. Prod. Commun.* **13**, (2018).

46. Zhou, M. *et al.* Antiviral butyrolactones from the endophytic fungus *Aspergillus versicolor*. *Planta Med.* **81**, (2015).

47. Zhou, G. *et al.* Prenylated p-Terphenyls from a Mangrove Endophytic Fungus, *Aspergillus candidus* LDJ-5. *J. Nat. Prod.* **83**, (2020).

48. Zhou, M. *et al.* Aspergillines A-E, highly oxygenated hexacyclic indole-tetrahydrofuran-tetramic acid derivatives from *Aspergillus versicolor*. *Org. Lett.* **16**, (2014).

49. Cheng, Z. *et al.* Versiquinazolines A-K, Fumiquinazoline-Type Alkaloids from the Gorgonian-Derived Fungus *Aspergillus versicolor* LZD-14-1. *J. Nat. Prod.* **79**, (2016).
